# Population genomics reveal apomixis in a novel system: uniclonal female populations dominate the tropical forest herb family, Hanguanaceae (Commelinales)

**DOI:** 10.1093/aobpla/plaa053

**Published:** 2020-09-24

**Authors:** Matti A Niissalo, Jana Leong-Škorničková, Otakar Šída, Gillian S Khew

**Affiliations:** 1 Singapore Botanic Gardens, National Parks Board Singapore, Singapore, Singapore; 2 Department of Biological Sciences, National University of Singapore, Singapore, Singapore; 3 Department of Botany, National Museum, Cirkusová, Prague, Czech Republic

**Keywords:** Apomixis, chromosome count, clonality, Commelinales, ddRADseq, genome size, *Hanguana*

## Abstract

The abundance of apomixis in tropical plant genera is poorly understood, and this affects the understanding of speciation and evolution. Hanguanaceae is a tropical monogeneric, dioecious plant family. All but two species are solitary herbs with no capability to spread vegetatively. Viable seeds are often produced when males have not been observed. Our aim was to investigate the presence of apomixis in *Hanguana*. We used reduced representation genomics to study phylogenetics and genetic variability in all populations of *Hanguana* in Singapore. We measured genome sizes and estimated ploidy levels in 10 species. Almost all taxa tested were genetically uniform (uniclonal) regardless of the extent of their distribution. The distribution of single clones over distinct localities supports our hypothesis of apomictic reproduction. Only one sexually reproducing native species was detected. Triploid and pentaploid states support our hypothesis that the type of apomixis in *Hanguana* is gametophytic. Population genomics tools offer a quick and cost-effective way of detecting excess clonality and thereby inferring apomixis. In the case of *Hanguana*, the presence of male plants is a strong indicator of sexual reproduction, whereas genome triplication is indicative of apomictic reproduction.

## Teaser

We used reduced representation genomics to observe population diversity in the dioecious herb genus *Hanguana* in Singapore. Our results suggest that most of the studied species reproduce exclusively via apomictic seeds, and form populations that are composed of single, widely distributed clones. This explains a lack of records of male plants in the area. The relationships of apomictic clones to sexual populations, for example possible hybridization, require further study. Apomictic clones of *Hanguana* are associated with uneven ploidy levels.

## Introduction

Clonal reproduction is a common feature in plants (Stuefer *et al.* 2002; Aarssen 2008). In some plants, the production of clonal, apomictic seeds happens in place of sexual reproduction. This specialized form of clonal reproduction involves the development of seeds from unreduced gametophytic tissue, or from other fruit tissue, resulting in clonal offspring (Koltunow 1993). The evolution and inheritance of gametophytic apomixis is complex and involves genes required for the production of unreduced gametes, parthenogenesis (the formation of embryos without the fertilization of female gametes) and the development of an endosperm with or without fertilization (e.g. Grossniklaus *et al.* 2001; van Dijk and Bakx-Schotman 2004).

Over a hundred tropical plant genera are included in the apomixis database (http://www.apomixis.uni-goettingen.de) but little is known about the prevalence of apomictic species within these genera. Hojsgaard *et al.* (2014) noted that: (i) the number of apomictic genera reduces from the tropics to higher latitudes; (ii) the proportion of apomictic species to the total flora could be lower in the tropics than in temperate areas; (iii) the available data are insufficient to back this up. Our current understanding about the evolution of apomixis is largely based on temperate species. The apomixis database includes only one family of commelinid monocots, i.e. the grasses (Polaes: Poaceae), but none in the order Commelinales which includes Hanguanaceae.

The detection of apomixis in tropical floras is hampered by a general lack of knowledge of the reproductive systems of tropical plants. Apomixis can be detected by the isolation of female reproductive organs, but this is technically complicated and is not routinely done. Apomictic species can also be identified by the over-representation of multilocus genotypes within a species (Tibayrenc *et al.* 1991). A multilocus genotype is a unique combination of alleles across two or more loci. In plants, where vegetative spread can result in similar observations, the over-represented multilocus genotypes must be observed in geographically distinct populations in order for apomixis to be inferred.

Morphologically similar apomictic clones may originate repeatedly from predominantly sexually reproducing populations or via hybridization between sexual and apomictic populations (Richards 1973; Bayer 1990a). There may be barriers to sexual reproduction of apomictic plants (e.g. genes and ploidy levels that affect meiosis, and unreduced gametes) but the barriers can be overcome and result in occasional sexual reproduction (Asker 1980; Hojsgaard 2018). Morphologically distinct apomictic plants are nearly always the result of sexual reproduction (van der Hulst *et al.* 2003). Hörandl and Paun (2007) found that the number of genetically distinct individuals in an apomictic population is much higher if sexually reproducing individuals are also present in that population. This results in the genotypic variation between apomictic individuals being almost equivalent to that of sexually reproducing individuals.

*Hanguana* is the only genus in the monocot family, Hanguanaceae. The genus is of little economic value except for one species that is commonly cultivated as an ornamental species (*Hanguana anthelminthica*). Until the 1980s, the genus was considered to include only one highly variable species, *H. malayana* (Backer 1951). *Hanguana malayana* is now considered to be endemic to a narrow range in Peninsular Malaysia (Leong-Škorničková and Niissalo 2017). The genus currently includes 18–19 described species that are morphologically well defined. In Singapore, *Hanguana* are restricted to primary forests. Five native species have been recorded: *H. neglecta*, *H. nitens*, *H. podzolicola*, *H. rubinea* and *H. triangulata*; the last two are considered endemic to Singapore (Niissalo *et al.* 2014; Leong-Škorničková and Boyce 2015; Niissalo and Leong-Škorničková 2017). Many species remain to be described and the species number likely exceeds 50 globally (Leong-Škorničková and Boyce 2015). Closely related *Hanguana* species are difficult to identify without fruits, and fruiting is rare. We therefore suspect that there may be more species even in Singapore than currently reported, i.e. the genus may have cryptic species diversity. Many species are narrow endemics, with the notable exception of two stoloniferous species, *H. anthelminthica* and *H. nitens*. *Hanguana anthelminthica* is a floating or marginal aquatic species found from India in the north-west to the Pacific islands in the south-east, while *H. nitens* is a terrestrial species confined to forested streams in Peninsular Malaysia and Singapore. The other described species are solitary forest herbs that only reproduce by seeds.

*Hanguana* species are dioecious. Male plants have so far been recorded in nine species, and they are rare in herbaria. This could be due to the short lifespan of male inflorescences (Leong-Škorničková and Boyce 2015). *Hanguana* fruits mature slowly and are prominently displayed for 6–10 months, whereas male flowers are ephemeral, lasting a few days to a few weeks.

Herbarium records that began in the 1820s show that, in Singapore, males have only been collected from *H. nitens* (Leong-Škorničková and Boyce 2015). Despite extensive surveys, we have never seen male individuals of any other species, even though all species in Singapore have a reliable seed set. The fruits of all species have a prominent endosperm. It is not known if the endosperm originates from gametophytic tissue. We have confirmed the viability of the seeds of all species *ex situ*. The distribution of almost all native species in Singapore can only be explained by seed dispersal: all native species except *H. nitens* are non-stoloniferous, and their distribution cannot result from vegetative expansion.

We use double-digest restriction site-associated DNA sequencing (ddRADseq) to detect clones in *Hanguana*. Identifying widespread clones in the landscape can offer strong evidence for apomixis when it is not accompanied by vegetative spread. The use of molecular methods for the detection of apomixis is particularly relevant where flowering is sporadic and the monitoring of fruit development is not feasible. We hypothesize that: (i) many *Hanguana* species are composed of only female individuals that reproduce apomictically, and that this can be detected by an over-representation of clonal plants. We use pairwise comparisons of genetic markers to distinguish clones and to test if clonal plants are over-represented within populations; (ii) apomictic plants originating from gametophytic apomixis have odd-numbered ploidy levels. To test this, we explore genome sizes, chromosome counts and relative allele copy numbers; and (iii) there are still undescribed cryptic taxa in Singapore. To test this, we estimate genetic distances between samples. However, we make no attempts to describe taxa based on molecular results, alone.

We also reconstruct the phylogeny of *Hanguana* in Singapore, with the aim of understanding the distribution of sexual reproduction in a phylogenetic context. We propose a pragmatic species concept in this genus and highlight areas that need to be better understood to improve taxonomic treatments in *Hanguana*.

## Materials and Methods

### Workflow

**Figure 1.**
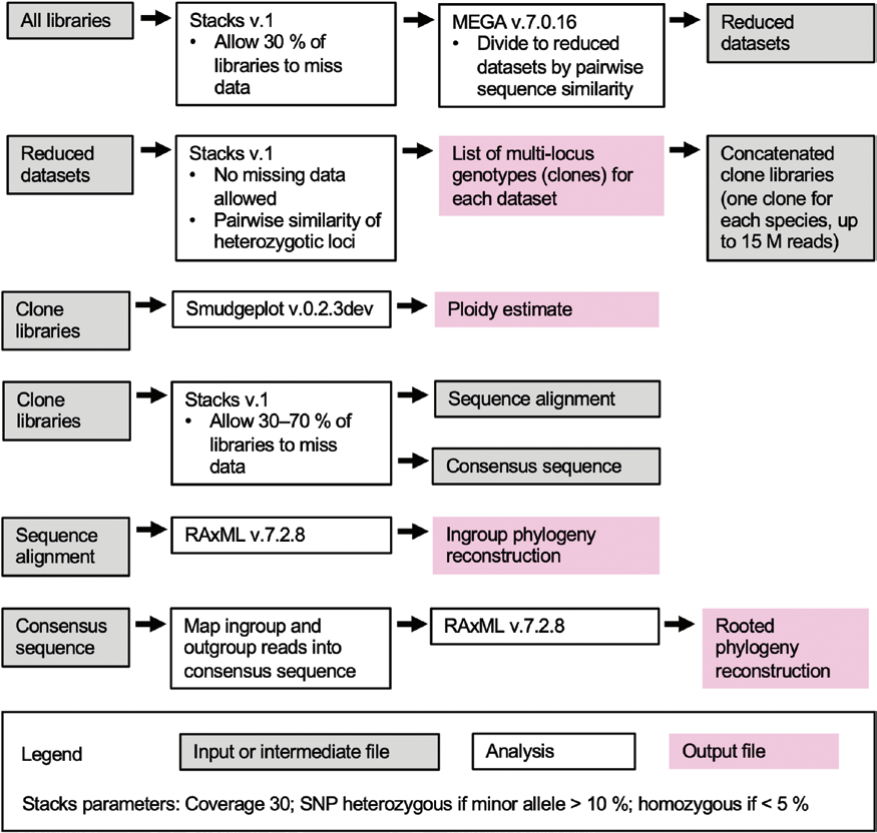
Work flow of sequence analyses downstream from demultiplexing and sequence quality control.

The workflow in Fig. 1 shows the sequence of the genetic analyses that we conducted, and the data sets used for each analysis. As the species identities of some sterile samples were unclear at the beginning of the analyses, the final workflow was completed after the initial genotypes and species identities of all samples were known, and contaminated libraries were removed.

### Sample collection

**Figure 2.**
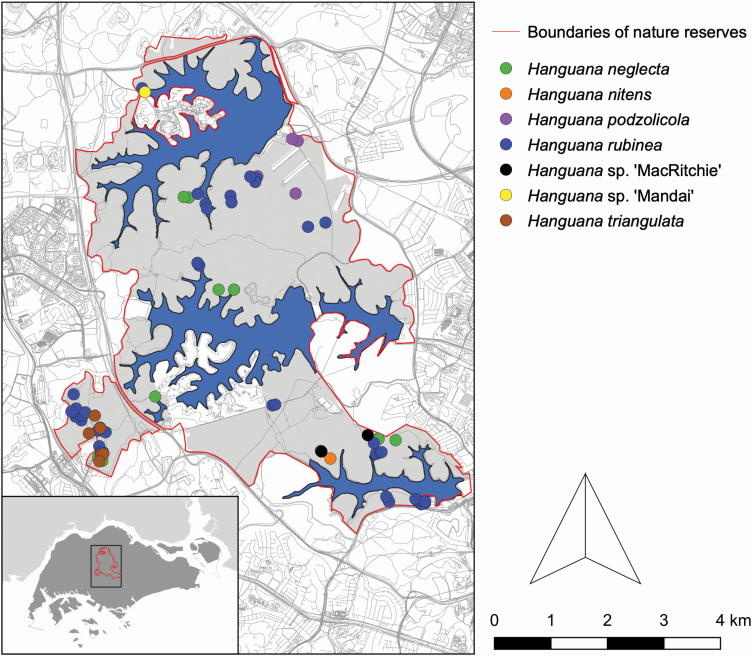
Collection localities of species native to Singapore.

We surveyed the populations of *Hanguana* in primary forests in two nature reserves in Singapore, Bukit Timah Nature Reserve (BTNR) and Central Catchment Nature Reserve (CCNR). In total, we collected tissue from all species in all 75 distinct localities in Singapore (‘locality’ here refers to a field collection that was georeferenced. While some localities were close to each other, minimum *c.* 10 m distance, none were close enough to allow vegetative expansion between localities in solitary species (see Fig. 2; **see**  **Supporting Information—Table S1**). A total of 130 tissue samples were dried using silica gel (Wilkie *et al.* 2013). While not all individuals (individual is here defined as a vegetatively independent unit of a plant) were sampled in larger subpopulations, the sampling included a substantial portion of the individuals of all native species (*H. neglecta*, *H. nitens*, *H. podzolicola*, *H. rubinea* and *H. triangulata*) and all samples that could not be identified. Three individuals were not seen in fruit and could not be reliably placed in any species; they are here given locality names: *H.* sp. ‘MacRitchie’ (two individuals) and *H.* sp. ‘Mandai’ (one individual). In addition, material from three non-native species, *H. anthelminthica*, *H. corneri* and *H. fraseriana*, was included to aid the phylogenetic reconstruction, adding to a total of 137 samples (Table 1). Duplicate libraries (total 20) were made of all species, using the same biological samples, where the error rates between libraries needed to be controlled. Here, ‘Duplicate library’ refers to a second DNA library and sequencing reaction of a biological sample.

**Table 1.** Biological samples and key results of the genomic analyses. *Clonality could not be tested, sexual system inferred.

	Number of samples (+ duplicates)	Number of localities	Native status in Singapore	Growth habit	Number of clones detected	Males recorded in Singapore	Sexual system detected	Est. chrom. numbers (counts)	Ploidy level	2C genome size in pg	1Cx genome size in pg
*Hanguana anthelminthica*	5	NA	Non-native	Stoloniferous	2	NA	Sexual*	80–96 (5)	4*x*	2.290	0.573
*Hanguana corneri*	1	NA	Non-native	Solitary	1	NA	Sexual*	40–48 (2)	2*x*	1.493	0.747
*Hanguana fraseriana*	1	NA	Non-native	Solitary	1	NA	Apomictic*	NA	3*x*	2.099	0.700
*Hanguana neglecta*	23 (7)	11	Native	Solitary	1	N	Apomictic	120–144 (5)	5*x*	3.561	0.712
*Hanguana nitens*	12 (3)	1	Native	Stoloniferous	8	Y	Sexual	40–48 (3)	2*x*	1.276	0.638
*Hanguana podzolicola*	8 (1)	5	Native	Solitary	1	N	Apomictic	60–72 (5)	3*x*	2.050	0.683
*Hanguana rubinea*	75 (4)	47	Native	Solitary	1	N	Apomictic	60–72 (3)	3*x*	2.027	0.676
*Hanguana triangulata*	9 (3)	8	Native	Solitary	1	N	Apomictic	NA	3*x*	2.001	0.667
*Hanguana* sp. ‘MacRitchie’	2 (1)	2	Native	Solitary	1	N	Apomictic	NA	3*x*	1.990	0.663
*Hanguana* sp. ‘Mandai’	1 (1)	1	Native	Solitary	1	N	Apomictic*	NA	3*x*	1.995	0.665
Total	137 (20)	75			18						

Sampling included individuals from the holotype localities of *H. fraseriana*, *H. neglecta*, *H*. *rubinea* and *H. triangulata*, and the neotype locality of *H. podzolicola*. The sample of *H. corneri* included here is from a paratype (Leong-Škorničková and Boyce 2015).

Herbarium vouchers were collected from flowering plants, but sterile vouchers were also collected from plants that were distinct in vegetative morphology. The herbarium vouchers were lodged at SING with the HAN-series numbers used in this paper.

### Chromosome counts and genome sizes

Chromosome spreads were prepared from fresh, actively growing roots. Roots were treated for 4–5 h at 16 °C in a saturated solution of paradichlorobenzene, or for 2 h at 4 °C in tap water, and fixed in Farmer’s Liquid at 4 °C overnight. The treated roots were then stored in 70 % EtOH at 4 °C for up to several weeks. The roots were softened for 20–60 s at 60 °C, in one part 1 N hydrochloric acid and three parts 45 % glacial acetic acid. The roots were stained in 1 % acetic orcein for 20–120 min. The roots were then placed on a microscope slide in 1 % lactopropionic orcein, covered with a cover slip and squashed. Photographs of cells used for counting were retained. In addition, recorded chromosome numbers of *Hanguana* were obtained from published literature (Bayer *et al.* 1998; Rudall *et al.* 1999; Hanson *et al.* 2003).

Holoploid genome sizes, equivalent to nuclear DNA 2C-values, were estimated using propidium iodide flow cytometry (FCM). Sample preparation generally followed the simplified procedure originally described by Otto *et al.* (1990). About 1 cm^2^ of young and intact fresh leaf tissue and an internal standard were co-chopped in a sandwich-like arrangement with a sharp razor blade in 0.5 ml of ice-cold Otto I buffer (0.1 M citric acid, 0.5 % Tween 20). The nuclear suspension was filtered through a nylon mesh with a 42-mm pore size. After incubating for 10 min at room temperature, 1 mL of Otto II buffer (0.4 M Na_2_HPO_4_·12H_2_O), supplemented with propidium iodide at a final concentration 50 µL·mL^−1^, RNase IIA (50 µL·mL^−1^) and 2-mercaptoethanol (2 µL·mL^−1^), was added. The samples were incubated for 10 min at room temperature, after which a fluorescence intensity of 3500 particles was recorded on a Partec Cyflow instrument (Partec GmbH, Münster, Germany) equipped with a 532-nm solid-state laser (Cobolt Samba 100 mW, Cobolt, Sweden). Each plant was re-analysed at least three times on different days and only histograms with peaks of approximately the same height were accepted. If the day-to-day variation (max/min value) exceeded 2 %, a further measurement was added until the standard error of mean decreased to under 1 %. *Solanum pseudocapsicum* (2C = 2.61 pg) was selected as a primary internal reference standard for all species. The genome size of *S. pseudocapsicum* was calibrated against our primary standard, *Bellis perennis* (2C = 3.42 pg; based on 13 replications spread over several days). The genome size of the primary standard has been established by calibration against *Pisum sativum* with 2C = 8.76 pg (Greilhuber *et al.* 2007; based on 14 replications). In total, 38 samples were measured.

### DNA extraction and library preparation

We used the DNeasy Plant Mini Kit (Qiagen) following the manufacturer’s protocol with two additional washes with 70 % EtOH. The eluted DNA was further purified with SeraMag Magnetic Carboxylate-Modified Microparticles (Thermo Scientific) and washed twice with 85 % EtOH.

Libraries were prepared with the methods and oligonucleotide sequences provided by Peterson *et al.* (2012). We used indices 1 and 2 out of the standard i5 Illumina multiplexing read indices listed in Peterson *et al.* (2012). Combined with the novel barcodes provided by Peterson *et al*., we acquired a total of 96 unique barcode combinations. We digested the DNA with *Ape*KI and *Pst*I. For fragment size selection (275–575 bp), we used SeraMag Magnetic Carboxylate-Modified Microparticles (Thermo Scientific) diluted to 5 %. For PCR, we used Phusion High-Fidelity DNA Polymerase (New England Biolabs), cycled for 12 cycles at an annealing temperature of 64 °C. We conducted the size selection and PCR separately for each sample, and tested their molarity and size range before pooling. No prior information on the genome sizes of the species and the cut frequency resulting from the restriction enzyme digestion was available. Therefore, all samples were multiplexed in the same molar concentrations, and deep sequencing was used to increase the number of available loci (*c*. 5 M reads per library, or 475 M bases).

### Sequencing, quality filtering and single-nucleotide polymorphism identification

The pooled libraries were sequenced by AITBiotech (Singapore), using Illumina HiSeq 2500 with V4 reagents. The read length was 100 bases, of which five bases were in-read index sequences. The index sequences were trimmed after demultiplexing. The samples were demultiplexed using the *process_radtags* function in Stacks (Catchen *et al.* 2013). Reads with a Phred score lower than 10 were discarded, giving a minimum confidence of 90 % for base calls with a sliding window of 15 % of the length of the sequence, i.e. average Phred score was counted for each 15 % portion of the sequence. Only full-length sequences (95 bases after trimming barcodes, with a minimum Phred score of 10 throughout the sliding windows) were retained.

We conducted the *de novo* sequence assemblies and identification of single-nucleotide polymorphisms (SNPs) using Stacks (Catchen *et al.* 2013). Two base differences were allowed between homologues, and only a single SNP was used per stack. Only sequences with a minimum stacks depth of 30 reads were retained. A minor SNP was called if it was present in >10 % of reads. A locus was discarded if a minor SNP was present in 5–10 % of reads. Loci with minor SNPs present in fewer than 5 % of the reads were considered homozygous and the minor SNPs were treated as error.

### Separation of samples to reduced data sets

We initially collected all SNPs with no missing data across all 157 libraries. This resulted in only 150 SNPs. As the number of SNPs was low, we relaxed the parameters and allowed missing data for 30 % of samples for each locus. This resulted in 24 557 SNPs. The overall comparison of SNP similarity was done using MEGA 7.0.16 (Kumar *et al.* 2016). We computed pairwise distances between all samples using the no variance estimation option, the *p*-distance method and including transitions and transversions in the substitutions. Uniform rates were assumed and pairwise deletion was used for missing data. Using the least ambiguous cut-off point of 99.78 % sequence similarity, it was possible to identify four groups that were genetically very similar. Data sets corresponding to these four groups were created, including all SNPs, and no missing data were allowed for any sample.

### Genotyping and the identification of clonal individuals

**Figure 3.**
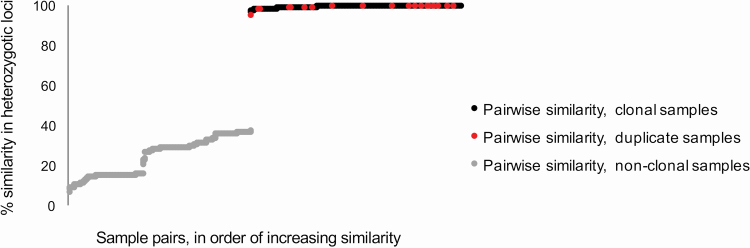
Identification of clones through pairwise comparisons. The values shown are percentage similarities between heterozygotic loci of all samples of *Hanguana* from the large forest species clade, arranged in order, from low to high. Similarity values between duplicate samples (DNA extracted from the same tissue sample) are in red. We considered sample pairs with higher than 85 % pairwise similarity to be of the same clone (black), and lower than 85 % to be of different clones (grey).

The discovery of clonal samples was done by a pairwise comparison of heterozygotic SNPs, i.e. each heterozygotic SNP in a sample was compared to its paired locus in another sample, and the percentage of identical matches was recorded. We assumed that different clones of sexually reproducing plants would differ in their heterozygotic SNPs even between siblings, as occurs in most natural situations (the term ‘clone’ here refers to a confirmed genetic entity, i.e. to genetically identical organism(s), consisting of a single or multiple individuals, produced asexually after a single, shared sexual event). The method closely followed Niissalo *et al.* (2018), with the addition of known duplicates to confirm the methodological error rate. We observed a clear threshold in pairwise similarity between sample pairs. Based on this threshold and the observed values between known duplicates, sample pairs were identified as belonging to the same clone when they shared at least 85 % of heterozygotic loci (in all data sets 88.91–100.00 %, average 99.17 %; Fig. 3). We have made available a script for comparison of the heterozygotic loci from the Stacks output in genepop format (https://github.com/mattiniissalo/Genepop_clonality_script).

Further quality control was done on a few samples that had unusual values in the pairwise analyses (pairwise similarities were lower than 50 % compared with all of the widespread clones). New libraries were prepared to confirm if the low similarity was due to contamination or sequencing error. Some of these libraries were confirmed to belong to new clones, and others belonged to the widespread clones when re-sequenced. One duplicate sample of *H. nitens* with excessive heterozygosity was removed, as it probably had been contaminated with DNA from another clone of the same species. A new duplicate library was prepared to replace it. The entire work flow was completed from the beginning after the problematic libraries had been removed.

We mapped reads of the original libraries to the plastome of *H. anthelminthica* (GenBank ID KT312930; Barrett *et al.* 2015) to determine what may have caused the poor quality of the libraries that were later confirmed to belong to widespread clones. A few ddRADseq reads mapped to the plastome successfully, and cross-sample contamination was detected by the presence of plastome reads from multiple species.

### Phylogenetic reconstruction

Three data sets were prepared for phylogenetic reconstruction with one clone per species. For two species, several clones were available (*H. anthelminthica* and *H. nitens*). In these cases, the clone with most available sequence data was chosen:

- Allowing missing data for 0 % of species provided 5018 SNPs (723 parsimony-informative).
- Allowing missing data for a maximum of 50 % of species per locus provided 60 565 SNPs (6867 parsimony-informative).
- Allowing missing data for a maximum of 70 % of species per locus provided 83 879 SNPs (7529 parsimony-informative).

Each taxon was represented by a single clone. Approximately 15 M reads were used per taxon, although we had fewer reads available for some species. The libraries used, and the amount of data per library can be seen in **Supporting Information—Table S1**. Entire fragments (95 bases per fragment) were used in a maximum likelihood analysis, while only the SNPs were used for a heuristic parsimony search.

Phylogenetic trees were reconstructed using parsimony in PAUP* 1.0a150 (Swofford 2002; variable loci were treated as polymorphisms), and maximum likelihood in RAxML 7.2.8 (Stamatakis 2006, setting -1 a –x 1 and 1000 bootstrapping iterations). The latter is incorporated in Geneious 9.0.5 (Biomatters, Ltd). Rooting was done at midpoint in FigTree 1.4.3 (available from http://tree.bio.ed.ac.uk/software/figtree/; accessed 3 May 2019). Due to a very low RAD allele overlap, outgroup samples (*Tradescantia zebrina*, *Pontederia cordata*, *Zingiber singapurense*, *Cyrtostachys renda*) did not provide any phylogenetically informative variation that could be used for rooting using the above methods. The following support values for trees were collected: (i) bootstrap with 50 replicates and jackknife support with 50 % deletion for parsimony (PAUP); (ii) bootstrap support for maximum likelihood (RAxML). We tested for the best substitution model for some data sets using jModelTest 2.1.10 (Darriba *et al.* 2012). Testing the substitution model for the largest data sets became computationally challenging and was abandoned. GTRGamma was always among the models with the lowest AIC (Aikike Information Criterion; none of the better models were incorporated in the RAxML version available). We therefore used the GTRGamma substitution model for all data sets. Changing the model to GTRGammaI did not affect the outcome of our analyses.

Further phylogenetic analyses were done with outgroups to create a rooted phylogenetic reconstruction. For this purpose, a sequence alignment was done by mapping sequence reads to a consensus of the ddRADseq output (all loci found in 30× coverage in at least 50 % of species, 42 685 loci). Mapping was done using the Geneious 9.0.5 read mapper, using medium–low sensitivity without gaps for the ingroup, and medium sensitivity without gaps for the outgroups. We used Geneious 9.0.5 for SNP calling. A minimum coverage of 10× was required to call loci, and only the most common nucleotides (present in 50 % of mapping reads or more) were called.

In addition to the ddRADseq libraries of the study samples, we used a whole-genome sequencing library of *Amischotolype hispida* (Commelinaceae; SRA: SRR7121777; reference Liu *et al.* 2019). Due to the short length of the reference reads (95 bases), the input sequences of *A. hispida* (100 bases) were split in the middle to two reads of 50 bases each to allow mapping to the ddRADseq loci (60.8 Gbases total). We relaxed the minimum coverage to three for *A. hispida*, and the mapper sensitivity was decreased to medium–low. Due to a low overlap seen in the other outgroups’ ddRADseq loci, this additional sample was required to firmly place Commelinaceae as a sister to Hanguanaceae and to resolve the basal relationships in the *Hanguana* included in this study.

The phylogeny was reconstructed using RAxML with the settings used for the ingroup phylogeny.

### Ploidy analysis

We estimated ploidy levels using demultiplexed reads in Smudgeplot v. 0.2.3dev (Ranallo-Benavidez *et al.* 2020), a kmer-based ploidy depth analysis software. We defined kmers as 90 bases (out of a total read length of 95 bases), as we did not expect much length variation in the homologous enzyme cut site-associated regions. We used KMC v. 3.1.0 (Kokot *et al.* 2017) to calculate kmers, and the parameters were defined as recommended in the Smudgeplot documentation. We expected that: (i) both kmers in a diploid locus should contribute equally (50 %) to the read depth; (ii) the minor kmer in a triploid locus should contribute 33 % to the read depth; and (iii) the minor kmer in a tetraploid locus should contribute 25 % to the read depth, etc. Only the *x*-axis of the output is informative, as the *y*-axis and the ploidy assignment of Smudgeplot is meaningless with ddRADseq data due to the non-normal distribution of sequence coverage. The same libraries were used as for the phylogenetic reconstruction detailed above.

 We also used relative SNP coverage to test if the inclusion of separate libraries of the same clone could affect the peaks of relative coverage of minor alleles (shown in the *x*-axis of a Smudgeplot graph). This test was done by using the coverage information for SNPs. Using SNPs instead of kmer pairs allowed us to increase the resolution of the peak position due to the increase in data available. We determined the SNP coverage by mapping reads to a reference sequence as described above for rooted phylogenies. A lower cut-off of 5 % was used for calling an allele, and a minimum total coverage of 20 was used. If the samples are clonal, we would expect that the peaks would not shift. As we expected, combining three libraries in any of the four possible combinations did not affect the positions of the peaks **[see**  **Supporting Information—Fig. S1****]**.

## Results

### Sequence data

The average sequencing depth of each library after demultiplexing and filtering (including the removal of all reads <95 bases) was 4.951 M reads (standard deviation 1.021 M) or 470.345 M bases (standard deviation 97.032 M). The size of each library is shown in **Supporting Information—Table S1**.

### Separation of samples to reduced data sets

Four groups, based on an overall sequence similarity of 99.78 % or higher, could be identified for population level analyses **[see**  **Supporting Information—Fig. S2****]**: (i) *H. anthelminthica*; (ii) *H. nitens*; (iii) small forest species (*H. corneri* and *H. neglecta*); and (iv) large forest species (*H. fraseriana*, *H. podzolicola*, *H. rubinea*, *H*. *triangulata*, *H*. sp. ‘Mandai’ and *H*. sp. ‘MacRitchie’).

The number of SNPs differed between each data set (*H. anthelminthica*: 6580 SNPs; *H. nitens*: 2848 SNPs; small forest species: 585 SNPs; large forest species: 1755 SNPs). The number of SNPs approximately correlates positively with the number of genotypes identified and negatively with the number of libraries included, as expected as no missing data were allowed.

### Genotyping and the identification of clonal individuals

Pairwise comparison of heterozygotic SNPs showed a clear cut-off point between clonal and non-clonal libraries; duplicate libraries and assumed clones matched in similarity. No sample pairs had a similarity between (46.17 and 88.37 %) in most data sets. A noticeably smaller, but overlapping cut-off was observed in *H. nitens* (72.04–90.22 %). Library pairs with a lower similarity in heterozygotic loci than these cut-off points were considered to belong to different clones, and those above the cut-off point were considered to belong to the same clone. For pairwise comparisons of heterozygotic loci in the large forest species, see Fig. 3. For a combined figure of comparisons in all data sets, **see**  **Supporting Information—Fig. S3**.

Our analyses detected 18 clones in the samples included in this study, including 14 clones native to Singapore. The number of recognized clones was very low in all native species except for *H. nitens* (Table 1). Of the clones identified, four were nursery grown seedlings of *H. nitens*, and four were collected from the only wild population of *H. nitens*. One clone was found for each of the other recorded native species (*H. neglecta*, *H. podzolicola*, *H. rubinea* and *H. triangulata*). The additional two clones that were detected (here referred to as *H*. sp. ‘MacRitchie’ and *H*. sp. ‘Mandai’) could not be reliably identified to species because they were not seen in fruit; these clones are similar to *H. triangulata* and *H. rubinea*. As pointed out by Leong-Škorničková and Boyce (2015) the identification of *Hanguana* heavily relies on fruit characters, and separating these two clones based on leaf morphology is unreliable. The remaining four clones were from non-native species (two clones of *H. anthelminthica*, and one clone each of *H. corneri* and *H. fraseriana*).

### Phylogenetic reconstruction

**Figure 4.**
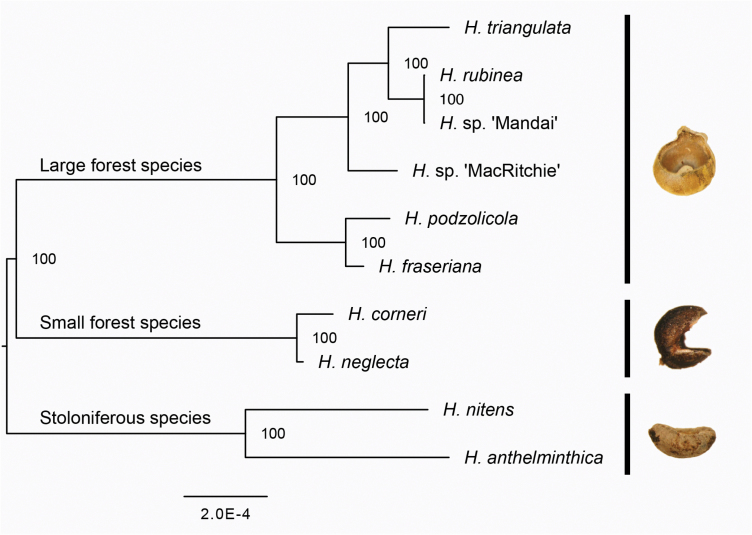
*Hanguana* phylogeny based on the maximum likelihood algorithm. Data used include SNPs missing from up to 70 % of taxa, with a maximum of one SNP per fragment. Support values shown are bootstrap values from 1000 replicates, rooted at midpoint. An example of seed morphology is shown for each named clade (seed of *H. anthelminthica* in the stoloniferous species clade, *H. neglecta* in the small forest species clade, *H. triangulata* in the large forest species clade).

We acquired fully resolved unrooted phylogenetic reconstructions. The tree topologies were identical in all analyses, and support values were high. Using the largest data set (70 % of species were allowed to miss data for any locus), all branches were fully supported in all analyses (Fig. 4). We rooted the tree at midpoint, resulting in the same topology as the rooted trees, described in the next paragraph.

The outgroup-rooted phylogeny provided a fully supported topology for the base of the tree **[see**  **Supporting Information—Fig. S4****]**. The two stoloniferous species, *H. anthelminthica* and *H. nitens*, form a clade that is sister to all the solitary, non-stoloniferous species in this study. The solitary species are divided into two major clades based on overall plant size and seed morphology; the large forest species with bowl-shaped seeds with a small seed appendage and the small forest species with ellipsoid seeds with a wedge-shaped ostiole.

### Chromosome counts, genome sizes and ploidy levels

As reported by Bayer *et al.* (1998), the chromosomes of *Hanguana* are very small, numerous and very difficult to count exactly. The roots are also slow-growing, with few cells in mitosis. We were unable to acquire precise counts in spite of exhaustive efforts. Chromosome numbers varied from ~40–48 to ~120–144. We therefore provide estimates (given as a range) for six species for which adequate preparations could be made (Table 1). Even within these samples there were considerable differences in preparation quality **[see**  **Supporting Information—Fig. S5****]**. The published counts in *Hanguana* (none identified to species) are 2*n* = 48, 72, 90, over 90 and *c.* 140 (Bayer *et al.* 1998; Rudall *et al.* 1999; Hanson *et al.* 2003). The chromosome counts we observed and those found from literature fit well with haploid counts of 20, 22 or 24. We therefore consider *Hanguana* species with ~40–48 chromosomes functionally diploid, those with ~60–72 functionally triploid, those with ~80–96 functionally tetraploid and those with ~120–144 functionally pentaploid or hexaploid.

**Figure 5.**
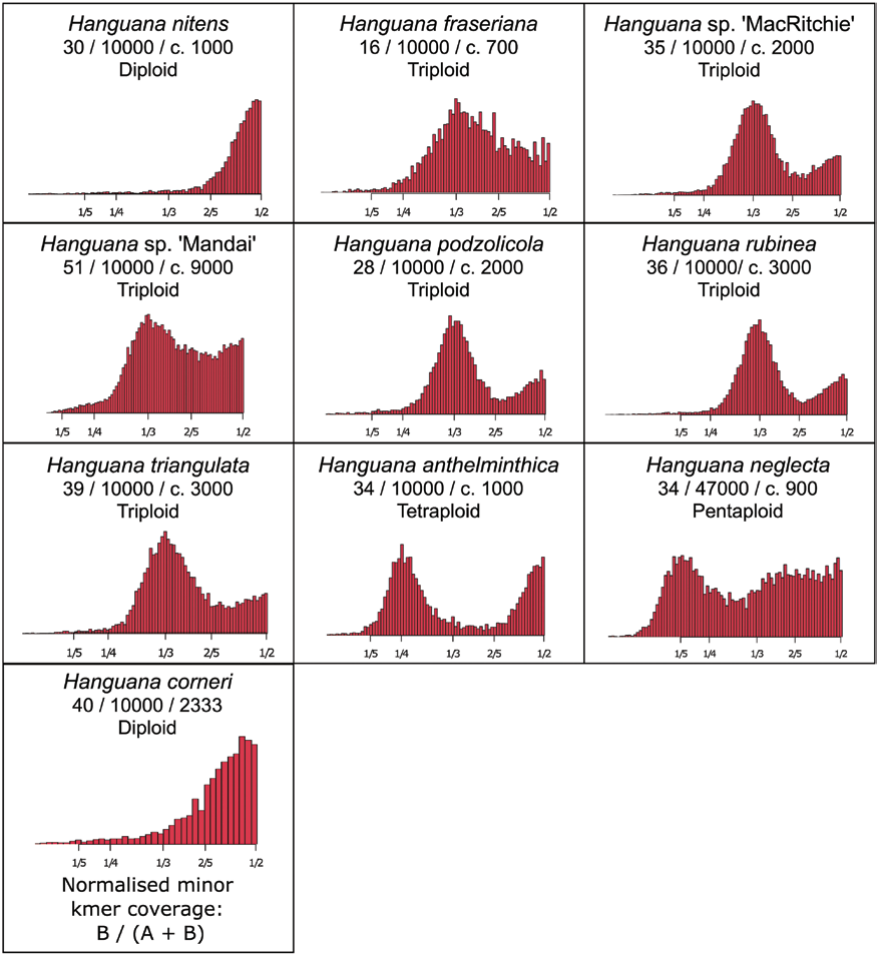
Smudgeplot analyses, showing normalized kmer pair ratios and estimated ploidy levels. *Y*-axes are not shown because the 1n coverage does not have a normal distribution in ddRAD sequencing data. The ratios for *H. corneri* were calculated from Smudgeplot output files, as there was inadequate data for a full analysis for that species. Values provided for taxon are lower limit of sequencing coverage, upper limit of sequencing coverage, number of kmer pairs in the highest peak observed, respectively.

The ploidy levels inferred from the chromosome counts were supported by the kmer balance obtained from the Smudgeplot analyses (Fig. 5): *H. corneri* and *H. nitens* are diploids, *H. fraseriana*, *H. podzolica*, *H. rubinea*, *H. triangulata*, *H*. sp. ‘MacRitchie’ and *H*. sp. ‘Mandai’ are triploids, *H. anthelminthica* is a tetraploid and *H. neglecta*, which could have been interpreted as a pentaploid or a hexaploid based on chromosome counts, was confirmed to be a pentaploid by the Smudgeplot analysis (Fig. 5; Table 1).

**Figure 6.**
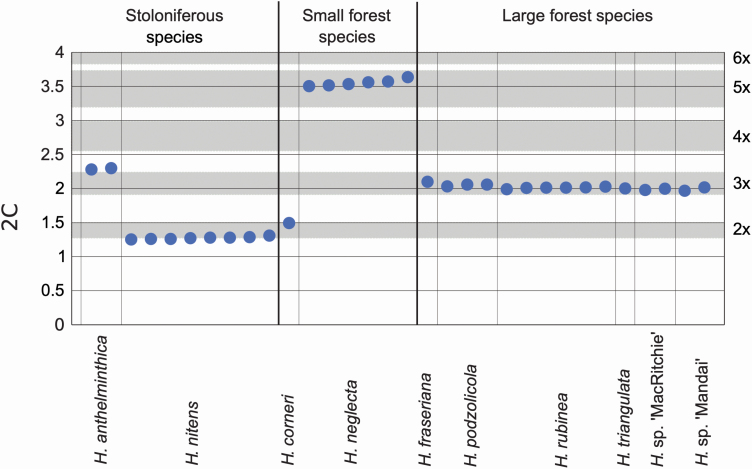
Distribution of 2C-values (DNA pg means) for the study samples. Grey bars indicate theoretical 2C-value ranges counted from the mean 1Cx-values of two diploid taxa, *H. nitens* and *H. corneri*. Note that the 2C-value of *H. anthelminthica* is lower than expected for a tetraploid (see Discussion). Bold vertical lines separate main clades.

Flow cytometric analyses yielded high-resolution histograms **[see**  **Supporting Information—Fig. S6****]**. The coefficients of variation (CVs) of G0/G1 peaks ranged from 1.99 to 5.69 % (median 3.39 %) for the *Hanguana* samples and from 1.73 to 4.89 % (median 3.03 %) for the reference standard. Day-to-day fluctuations caused by instrument instability or differences in sample preparation were negligible; the standard error of mean of repeated measurements on different days ranged between 0.18 and 1.38 %. The genome sizes varied in our data set from 2C = 1.276 pg in *H. nitens* to 2C = 3.561 pg in *H. neglecta*, representing a 2.79-fold range (Fig. 6; Table 1; **see**  **Supporting Information—Table S2**). The genome sizes of all samples are shown in Fig. 6. Intraspecific variation usually did not exceed 3 %, with the exception of *H. nitens* and *H. neglecta*, where it reached 4.3 and 3.6 %, respectively.

## Discussion

### Apomictic populations and the dynamics of apomixis

Four native species and two undescribed taxa (*H. podzolicola*, *H. neglecta*, *H. rubinea*, *H. triangulata*, *H*. sp. ‘MacRitchie’ and *H*. sp. ‘Mandai’) are each comprised of a single clone. This suggests that they are reproducing apomictically because vegetative spread of these solitary herbs cannot account for the geographic range that they have achieved. Furthermore, no males have been found during our surveys and in herbarium records, and their ploidy levels (triploid and pentaploid) suggest that they are not reproducing sexually. That each of these species is uniclonal is likely related to their dieocious reproductive system. As the female plants cannot produce pollen, there is no opportunity even for occasional sexual reproduction. Only *H. nitens* shows a level of genetic variability suggestive of sexual reproduction. *Hanguana nitens* is also diploid, and the only species in Singapore for which males have been sighted.

Two non-endemic species (*H. podzolicola* and *H. neglecta*) are uniclonal in Singapore, but may have sexually reproducing populations in Peninsular Malaysia, where males have been reported for *H. neglecta* (Niissalo *et al.* 2014). Two endemic species *H. rubinea* and *H. triangulata*, and presumed endemic species *H*. sp. ‘Mandai’ and *H*. sp. ‘MacRitchie’, are uniclonal and solely female. Although it is likely that sexual reproduction is more common outside of Singapore, the low number of male sightings in other parts of the region suggests that apomixis plays an important role in the reproduction and speciation processes in the genus.

Case studies in *Antennaria rosea*, a dioecious taxon (classified as a single species) of which populations predominantly consist of female individuals, are relevant to the understanding of clonality in *Hanguana* from a geographic perspective. The populations of *A. rosea* have a high genotypic diversity at the centre of its range, where they also reproduce sexually, but in the extreme populations usually only a single genotype is known (Bayer and Stebbins 1987; Bayer 1990b; Bayer *et al.* 1990). It is possible that like *Antennaria*, new apomictic clones of *Hanguana* emerge regularly in populations with male plants (Bayer 1990a).

As *Hanguana* species are exclusively found in primary forests in Singapore, the lack of sexual plants in Singapore could be a result of forest loss across the island. It is possible that the extant species are relicts of previously more diverse, sexually reproducing populations, and that forest clearing has resulted in the loss of sexual populations and genetic diversity. The fragmented habitats could have favoured apomictic plants, resulting in contemporary genetic patterns. However, the very small number of clones observed suggests that Singapore was never a centre of diversity for *Hanguana* taxa.

The patterns of clonality observed are better explained by geography and dispersal ability, rather than by forest loss. Singapore is positioned at the southern tip of the Malay Peninsula and isolated from it by a narrow sea strait. This decreases the migration of both sexes, thus favouring apomictic plants, which only require a single migration event to establish. The dispersal ability of *Hanguana* has not been reported. The fruit morphology suggests that they are dispersed by birds (W. Stuppy, Millennium Seedbank, Royal Botanic Gardens, Kew, pers. comm.). Using camera traps, we have observed birds consuming the fruits of *H. rubinea*. The dispersal ability could be affected by the life history of the specific bird dispersal agents, which requires further study.

Given their spread across suitable habitats in Singapore, the more widespread apomictic clones could have originated a very long time ago. Although herbaceous plants are often considered short-lived, this does not apply to *Hanguana*. We have observed extremely slow growth rates in non-stoloniferous *Hanguana* both *in situ* and *ex situ*, suggesting that large individuals with a long stem could be very old. Studies on Singapore Zingiberales suggest that primary forest plants rarely spread to new habitats in the current fragmented landscape (Niissalo *et al.* 2018). Intensive forest loss started in Singapore in 1819 and continued to early parts of the 1900s (Corlett 1992). We suspect that this is also true for *Hanguana*, and that the clones were likely present in their current range before forest loss.

The only sexually reproducing population, i.e. the only population of *H. nitens*, is very small in size, and consists of a few intermingled stoloniferous clones. Our *in situ* sampling detected four clones, but it did not include all the ramets in the population (the term ramet is here used for the sampled units of the two stoloniferous species, *H. anthelminthica* and *H. nitens*; in these species, it is not possible to discern vegetatively independent individuals, and distinct *ramets* (stems) were sampled from various parts of continuous populations). Based on the sampling density, and the clonality observed, we estimate that about half the clones present were detected. The four nursery grown seedlings, originating from the same infructescence, were each a distinct clone. We therefore have no evidence of apomixis in this species.

In non-native species, we were able to infer sexual systems without testing clonality by using the presence of males and the fact that gametophytic apomicts often have uneven ploidy levels (see discussion below). The single diploid individual of *H. corneri* included here was introduced to Singapore in the 1930s. It is male, and could therefore not be apomictic. The only individual of *H. fraseriana*, collected from Peninsular Malaysia, is a triploid female that had fully developed seeds, and is likely apomictic. However, males were observed in the population from which it was collected, and the original population is likely mixed. Two clones were found in *H. anthelminthica*, both of which were tetraploid. While this does not exclude apomictic reproduction, we have observed *H. anthelminthica* setting seed in the proximity of flowering male plants at Singapore Botanic Gardens. Further observations and more extensive sampling are required to confirm the presence of apomixis in this widely cultivated species.

### Chromosome counts, ploidy levels and genome size

Our observations of *Hanguana* chromosomes generally agree with previously published chromosome counts. High quality karyotypes are needed to determine the basic chromosome number(s), but our preparations were not of adequate quality. Our counts suggest that 1*x* = 20, 22 or 24. The lattermost value fits best with published counts, i.e. 2*n* = 48, 72, 90, over 90 and *c.* 140 (Bayer *et al.* 1998; Rudall *et al.* 1999; Hanson *et al.* 2003). The previous counts are difficult to relate to current taxa, as they predate most of the species descriptions in the genus and no vouchers are cited for most. 2*n* = 48 reported by Bayer *et al.* (1998) and Rudall *et al.* (1999) may refer to *H. bogneri*, a possibly sexually reproducing male plant, which was cultivated in Germany (Tillich and Sill 1999). Bayer *et al.* (1998) and Rudall *et al.* (1999) also report 2*n* ≥ 90 from Sri Lanka, where only *H. anthelminthica* is known to occur. A single count has been made from living material at the Royal Botanic Gardens, Kew (Hanson *et al.* 2003). This particular cultivated collection originated from Singapore, and is still present at Kew. We have confirmed the identity of this collection as *H. neglecta*. The reported value, ‘*c.* 140’, fits our observations, but could be an overestimate for a pentaploid taxon. We were unable to confirm the taxonomic identity of the rest of the material used in the above studies. The high basic number (1*x* = 20–24) suggests that the genus could be of paleopolyploid origin (Grant 1982).

We observed odd-numbered ploidy levels (3*x* or 5*x*) in all the uniclonal species. This is consistent with gametophytic apomixis (de Wet and Stalker 1974; Whitton *et al.* 2008). The triploid clones could have originated from diploid parents, by the fusion of an unreduced female gamete with a reduced male gamete (Asker 1980; Grimanelli *et al.* 2001). The ploidy of the parents of the pentaploid *H. neglecta* could not be deduced. Throughout this study, we observed a correlation between the ploidy levels, approximate chromosome counts and genome sizes.

Before the current study, there was only one published record of genome size, namely for *H. neglecta*, 2C = 3.29 pg (published as *H. malayana*; Hanson *et al.* 2003). Our 2C-value for *H. neglecta* is 8 % higher, which can be explained by the difference in method (Feulgen densitometry) and standard (*Hordeum vulgare* ‘Sultan’) used by Hanson *et al.* (2003).

Converting 2C-values to relative ploidy levels reveals some overall patterns in genome size evolution. The monoploid genome sizes are lowest in the clade of stoloniferous species (*H. nitens*, 0.638 pg, and *H. anthelminthica*, 0.573 pg). *Hanguana anthelminthica* has the lowest monoploid genome size of all, which could be due to gene loss that could have occurred through genome fragmentation of this tetraploid taxon. The phenomenon of genome downsizing in polyploid taxa is known to be common in vascular plants (Leitch and Bennett 2004) and is usually observed in evolutionarily older taxa, while in recently evolved complexes, the genome size ratio more accurately corresponds to the theoretical ratio of ploidy levels. We therefore suspect that *H. anthelminthica* may be an old tetraploid: we hypothesize that the genome duplication conferred an evolutionary advantage on this species to inhabit to a new niche (it is the only non-forest species and the only floating aquatic species in the genus) which in turn is the likely reason for the wide distribution (Leong-Škorničková and Niissalo 2017) of this species, much wider than any other species in the genus. The small forest species clade (*H. corneri*, 0.747 pg, and *H. neglecta*, 0.712 pg) and the large forest species clade (0.663–0.670 pg) are less variable in their monoploid genome size than the stoloniferous species clade.

Intraspecific genome size variation was negligible (lower than 4 %, i.e. within accepted instrumental fluctuation) except in *H. nitens* (4.3 %). In *H. nitens* the intraspecific variability was only slightly higher and most probably originated from instrumental fluctuation or sample quality. In no case can it be considered evidence of real intraspecific variation (Greilhuber 2005).

### Apomixis in Commelinales

Apomixis has been considered absent in Commelinales (Asker and Jerling 1992). The only record of apomixis in Commelinales involves a single wild-collected mutant of *Tradescantia spathacea* (Commelinaceae). This mutant exhibited interrupted meiosis, but was able to produce unreduced gametes that sometimes led to apomictic offspring in laboratory conditions. The species is otherwise sexually reproducing and apomictic reproduction has not been reported *in situ* (Garcia-Velazquez 1995). *Hanguana* is therefore the only genus in Commelinales in which apomixis is known in wild populations. A more thorough sampling of the genus and a better understanding of reproduction in the stoloniferous species clade are needed before we can determine if apomixis is limited to non-stoloniferous *Hanguana*, and how widespread it is outside of Singapore.

### Proposal of a species concept in *Hanguana*

The current state of knowledge on sexually reproducing populations of *Hanguana* is very limited. We therefore propose a pragmatic approach to species concepts to capture the diversity in the genus. Our proposal is similar to the species concept applied to *A. rosea* (Bayer 1990a). Sexual *Hanguana* can be treated according to the biological species concept. Apomictic plants should be classified by their relationship to sexual taxa, if this is known. If the apomictic clones have emerged from sexual populations of a single species, we would suggest that all the apomictic clones and their sexual relatives be treated together in that species. It is possible to acknowledge the taxonomic status of morphologically distinct and spatially isolated clones at the variety level. If, however, the apomictic plant is of known interspecies hybrid origin, it should be treated at the nothospecies level. Such clones could achieve a level of reproductive isolation and stability, but further hybridization events may occur. If only an apomictic clone of a morphologically distinct taxon is known and its relationship to sexual species cannot be determined, it is justifiable to recognize it as a distinct species (e.g. *H. rubinea* and *H. triangulata*, for which no sexual populations are known).

We strongly advocate that future descriptions of *Hanguana* should highlight key characteristics, which are of value to the application of the above species concept proposed above. The presence of male individuals indicates sexual reproduction, and therefore the biological species concept can be applied. To test clonality, genetic sampling should include several distinct individuals from different parts of a population. Genetic sampling from herbarium collections or cultivated plants often yield only single individuals. Based on our results, apomictic plants often have odd-numbered ploidy levels, with triploids being particularly common. This can be detected from single individuals. Methods such as Smudgeplot can be used to test ploidy levels using allele frequency as done here. Whole-genome sequencing would, additionally, give information of total kmer coverage, which is of value in ploidy estimation.

### Population genomics in the study of apomixis

Clonal diversity in apomictic species offers key insights into the dynamics and origins of apomictic species, populations and clones. Until recently, the knowledge of genetic diversity in apomictic plants has relied heavily on Random Amplification of Polymorphic DNA (RAPDs) and isozymes, which are hard to reproduce and may lead to underestimates of genotypic diversity (Nybom 2004; Hörandl and Paun 2007). More recently, studies using mainly Amplified Fragment Length Polymorphisms (AFLPs) and microsatellites have emerged (e.g. Lo *et al.* 2009; Loomis and Fishman 2009; Sartor *et al.* 2013; Majeský *et al.* 2015; Dias *et al.* 2018). Our method of detection of apomixis in naturally occurring populations is based on the detection of excess clonality in the lack of sexually reproducing individuals. It shares similarities to the methods of Tibayrenc *et al.* (1991) in that it uses multilocus genotypes to detect excess genotype dispersal. Only with an increased number of loci can we equate the genotypes with clones. Double-digest restriction site-associated DNA sequencing offers a standard method for the acquisition of genomic markers, and the identification of clones (Niissalo *et al.* 2018), that can be easily applied to case studies without prior knowledge of the genome or population genomic markers. In addition, this method has the particular advantage of allowing the use of dried tissue. The high number of loci that can be sequenced allows the identification of genotypes to the level of a clonal individual, as long as the methodological error rate is known. Based on the current results, the method also works reliably with polyploids—the identification of clones was unambiguous even for pentaploids. However, retrieving single alleles becomes more difficult as ploidy increases, and the parameters for allele calling may need to be relaxed and sequencing depth may need to be increased for high polyploids with large genomes.

## Supporting Information

The following additional information is available in the online version of this article—

**Table S1.** Geographic localities, sample names and sequencing depth of samples used in this study.

**Table S2.** Overview of holoploid (2C) genome sizes estimated in Singaporean *Hanguana* species.

**Figure S1.** Effect of using multiple libraries from different biological samples of *Hanguana neglecta* on relative contribution of a less frequent single-nucleotide polymorphism (SNP) to the total coverage. The position of the two observed peaks (20 and 40 %, consistent with a pentaploid plant) was not affected by the combination of multiple biological samples used, but the signal improved with increased data. The result was expected, as the samples are considered clonal.

**Figure S2.** Pairwise overall single-nucleotide polymorphism (SNP) similarity of all samples sequenced in this study.

**Figure S3.** Identification of clonal pairs in all four *Hanguana* data sets using only heterozygotic loci.

**Figure S4.** Rooted phylogenetic reconstruction, with the addition of outgroups.

**Figure S5.** Chromosome number variation in *Hanguana*.

**Figure S6.** Representative flow cytometric histogram documenting genome size determination.

plaa053_suppl_Supplementary_Materials

## Data Availability

Raw, demultiplexed sequence reads are available at the Sequence Read Archive (https://www.ncbi.nlm.nih.gov/sra) and can be accessed with a BioProject ID PRJNA663226. The sequence alignment with outgroup taxa is available in Figshare, at https://dx.doi.org/10.6084/m9.figshare.12950924.

## Notes

**Evolution & Diversity. Chief Editor**: Jeremy Beaulieu
