# Supplementary material for: Population genomics reveal apomixis in a novel system: uniclonal female populations dominate the tropical forest herb family, Hanguanaceae (Commelinales)
Source: AoB Plants. 2020 Sep 24;12(6):plaa053. doi: 10.1093/aobpla/plaa053 (PMC7653639; doi:10.1093/aobpla/plaa053)

Supporting Information Table 1

Geographic localities, sample names and sequencing depth of samples used in this study.

Cat* = used in the concatenated library for this species (one clone used per species)

**Locality name**	**Species**	**Latitude**	**Longitude**	**Notes**	**Sample/library name**	**Number of reads (95 bp each)**	**Notes**
Admiralty	*Hanguana anthelminthica*	N/A	N/A	Cultivated, Admiralty Park, Singapore	Adm	4,776,530	
JLS-3033	*Hanguana anthelminthica*	N/A	N/A	Cultivated, Singapore Botanic Gardens	JLS3033	4,306,572	
Lab14	*Hanguana anthelminthica*	N/A	N/A	Cultivated, Lab 14 in National University of Singapore	lab14	5,161,937	**Cat***
Uhall	*Hanguana anthelminthica*	N/A	N/A	Cultivated, University Hall in National University of Singapore	uhall1	5,137,178	**Cat***
					uhall2	4,860,977	**Cat***
HAN-04	*Hanguana corneri*	N/A	N/A	Introduced to Bukit Timah Nature Reserve, non-native, originally from holotype locality	HAN04_A1	5,353,726	**Cat***
FRI89123	*Hanguana fraseriana*	N/A	N/A	Holotype, Peninsular Malaysia	FRI89123	3,827,293	**Cat***
HAN-07	*Hanguana neglecta*	1.348°N	103.778°E	Holotype locality	HAN07_A15	7,697,831	
					HAN07_A23	4,076,634	
					HAN07_A24	4,379,835	
					HAN07_A29fi	4,504,841	**Cat***
					HAN07_A36	4,350,424	
					HAN07_A29Ase	4,532,439	Duplicate library **Cat***
					HAN07_A29Bse	4,490,305	Duplicate library **Cat***
HAN-26	*Hanguana neglecta*	1.356°N	103.830°E	Genome size only			
HAN-27	*Hanguana neglecta*	1.352°N	103.822°E		HAN27_A1	4,722,917	
HAN-50	*Hanguana neglecta*	1.394°N	103.792°E		HAN50_A1	4,818,006	
HAN-51	*Hanguana neglecta*	1.394°N	103.791°E		HAN51_A1	5,410,743	
					HAN51_A3	5,778,832	
					HAN51_A5fi	5,038,159	
					HAN51_A5se	3,929,961	Duplicate library
HAN-62	*Hanguana neglecta*	1.349°N	103.778°E		HAN62_F1	6,310,321	
HAN-74	*Hanguana neglecta*	1.352°N	103.825°E		HAN74_A02fi	4,811,189	
					HAN74_A4	4,296,995	
					HAN74_A07	4,014,339	
					HAN74_A09	3,906,500	
					HAN74_F1fi	3,489,894	
					HAN74_A02se	4,819,167	Duplicate library

					HAN74_F1se	4,122,636	Duplicate library
HAN-79	*Hanguana neglecta*	1.380°N	103.794°E		HAN79_A1A	5,953,411	
					HAN79_A14	5,938,049	
					HAN79_A1B	3,381,814	
HAN-81	*Hanguana neglecta*	1.376°N	103.797°E		HAN81_A1	4,703,423	
HAN-82	*Hanguana neglecta*	1.376°N	103.799°E		HAN82_A1	5,812,216	
HAN-84	*Hanguana neglecta*	1.359°N	103.787°E		HAN84_A1	5,542,920	
					HAN84_A7	4,266,381	
					HAN84_A14fi	3,733,493	Duplicate library
					HAN84_A14se	3,602,843	Duplicate library
HAN-01	*Hanguana nitens*	1.349°N	103.815°E		HAN01_f_A1	4,603,829	
					HAN01_f_A2	4,795,977	
					HAN01_f_A15	5,846,432	
					HAN01_f_B	4,176,961	
					HAN01_F8 (nursery seedling)	4,275,172	
					HAN01_F9 (nursery seedling)	4,124,334	
					HAN01_F10 (nursery seedling)	4,925,322	
					HAN01_F11A (nursery seedling)	5,133,245	
					HAN01_m_A1	4,989,316	
					HAN01_m_A2Afi	4,503,158	
					HAN01_m_A3	4,758,355	**Cat***
					HAN01_m_A4	4,748,957	**Cat***
					HAN01_F11B (nursery seedling)	4,467,823	Duplicate library
					HAN01_m_A2Bfi	5,678,338	Duplicate library
					HAN01_m_A2se	4,304,426	Duplicate library **Cat***
HAN-13	*Hanguana podzolicola*	1.394°N	103.803°E		HAN13_A1	5,624,822	
HAN-30	*Hanguana podzolicola*	1.393°N	103.803°E		HAN30_A1fi	5,415,994	
					HAN30_A1se	5,415,037	Duplicate library
HAN-75	*Hanguana podzolicola*	1.390°N	103.813°E	Genome size only			
HAN-76	*Hanguana podzolicola*	1.400°N	103.809°E	Neotype locality	HAN76_A01	6,118,276	**Cat***
					HAN76_A04	6,095,046	**Cat***
					HAN76_A06	6,327,300	**Cat***
					HAN76_A08	5,912,418	
					HAN76_A13	6,304,113	
HAN-86	*Hanguana podzolicola*	1.391°N	103.809°E		HAN86_A1se	4,468,359	
HAN-02	*Hanguana rubinea*	1.350°N	103.778°E		HAN02_A1	5,733,662	
					HAN02_A7	4,628,980	
					HAN05_A2	4,423,444	
HAN-05	*Hanguana rubinea*	1.350°N	103.778°E		HAN05_A3	4,636,227	
HAN-09	*Hanguana rubinea*	1.343°N	103.824°E		HAN09_A1	4,235,366	
HAN-10	*Hanguana rubinea*	1.342°N	103.824°E		HAN10_A1	5,554,844	
					HAN10_A2	4,229,357	
HAN-12	*Hanguana rubinea*	1.408°N	103.785°E		HAN12_A1	4,176,308	
					HAN12_A3	3,422,824	
					HAN12_A4fi	4,195,888	
					HAN12_A4se	5,186,070	Duplicate library
					HAN12_A5	4,071,842	
HAN-14	*Hanguana rubinea*	1.393°N	103.803°E	Holotype locality	HAN14_A04	5,929,248	
					HAN14_A12	6,115,861	
HAN-16	*Hanguana rubinea*	1.342°N	103.830°E		HAN16_A4	4,037,951	

HAN-17	*Hanguana rubinea*	1.342°N	103.829°E		HAN17_A1	3,794,761	
HAN-18	*Hanguana rubinea*	1.342°N	103.829°E		HAN18_A1	5,332,488	
					HAN18_F2	3,625,389	
HAN-19	*Hanguana rubinea*	1.342°N	103.828°E		HAN19_A1	5,321,934	
HAN-20	*Hanguana rubinea*	1.342°N	103.828°E		HAN20_A3	5,916,947	
HAN-23	*Hanguana rubinea*	1.343°N	103.824°E		HAN23_A2	5,238,254	
HAN-24	*Hanguana rubinea*	1.342°N	103.830°E		HAN24_A5	5,282,185	
HAN-29	*Hanguana rubinea*	1.386°N	103.811°E		HAN29_A1	4,549,669	
					HAN29_A2	4,704,028	
					HAN29_A3	5,784,632	
HAN-31	*Hanguana rubinea*	1.393°N	103.803°E		HAN31_A11	4,180,010	
					HAN31_A21	5,903,554	
					HAN31_A29	5,343,086	
HAN-33	*Hanguana rubinea*	1.394°N	103.802°E		HAN33_A3	7,981,029	
					HAN33_A8	5,376,453	
					HAN33_A17	7,001,262	
HAN-35	*Hanguana rubinea*	1.358°N	103.778°E		HAN35_A1	3,358,361	
HAN-36	*Hanguana rubinea*	1.353°N	103.778°E		HAN36_A2	4,203,786	
HAN-37	*Hanguana rubinea*	1.355°N	103.775°E		HAN37_A1	4,579,774	
HAN-39	*Hanguana rubinea*	1.355°N	103.774°E		HAN39_A3	5,023,344	
HAN-41	*Hanguana rubinea*	1.356°N	103.774°E		HAN41_A1	4,438,373	
					HAN41_F2	3,729,053	
HAN-42	*Hanguana rubinea*	1.356°N	103.775°E		HAN42_A1	4,815,130	
					HAN42_A3	4,673,396	
					HAN42_F2	3,996,454	
HAN-43	*Hanguana rubinea*	1.356°N	103.775°E		HAN43_A4	3,808,402	
					HAN43_F1	3,711,105	
HAN-45	*Hanguana rubinea*	1.357°N	103.774°E		HAN45_A1	4,143,189	
HAN-46	*Hanguana rubinea*	1.356°N	103.776°E		HAN46_A1	5,627,105	
HAN-48	*Hanguana rubinea*	1.356°N	103.774°E		HAN48_A1	4,677,620	
HAN-49	*Hanguana rubinea*	1.359°N	103.774°E		HAN49_A1	4,223,499	**Cat***
HAN-52	*Hanguana rubinea*	1.350°N	103.822°E		HAN52_A1	5,999,749	**Cat***
					HAN52_A3	6,589,488	**Cat***
					HAN52_A4	6,535,444	
HAN-53	*Hanguana rubinea*	1.350°N	103.822°E		HAN53_A1	5,397,478	
HAN-54	*Hanguana rubinea*	1.350°N	103.823°E		HAN54_A1	4,981,566	
					HAN54_F1	4,671,225	
HAN-55	*Hanguana rubinea*	1.350°N	103.823°E		HAN55_A1	4,783,510	
HAN-56	*Hanguana rubinea*	1.357°N	103.805°E		HAN56_A1	4,849,901	
HAN-57	*Hanguana rubinea*	1.357°N	103.806°E		HAN57_F1	5,270,801	
HAN-58	*Hanguana rubinea*	1.396°N	103.799°E		HAN58_F1	4,349,795	
HAN-59	*Hanguana rubinea*	1.389°N	103.795°E		HAN59_A1	3,842,024	
HAN-60	*Hanguana rubinea*	1.399°N	103.794°E		HAN60_A1	5,006,234	
HAN-61	*Hanguana rubinea*	1.389°N	103.795°E		HAN61_A1fi	5,051,345	
					HAN61_A1Ase	4,189,106	Duplicate library
					HAN61_A1Bse	4,862,305	Duplicate library
HAN-64	*Hanguana rubinea*	1.350°N	103.778°E		HAN64_A1	4,447,526	
HAN-65	*Hanguana rubinea*	1.353°N	103.779°E		HAN65_A1	6,085,927	

HAN-66	*Hanguana rubinea*	1.354°N	103.778°E	Mixed population	HAN66_A2	5,050,196	
HAN-70	*Hanguana rubinea*	1.350°N	103.778°E		HAN70_A08	5,862,077	
					HAN70_A10	5,681,073	
HAN-73	*Hanguana rubinea*	1.351°N	103.822°E		HAN73_A1	3,291,546	
HAN-77	*Hanguana rubinea*	1.380°N	103.794°E		HAN77_A1	4,949,489	
					HAN77_A3	3,345,060	
HAN-78	*Hanguana rubinea*	1.380°N	103.794°E		HAN78_A1	4,658,945	
					HAN78_A3	5,037,166	
					HAN78_A5	3,679,299	
HAN-85	*Hanguana rubinea*	1.407°N	103.785°E	Mixed population	HAN85_A2	4,708,592	
					HAN85_A5	7,116,946	
					HAN85_F1	7,192,269	
					HAN85_F2	9,840,187	
HAN-87	*Hanguana rubinea*	1.390°N	103.799°E		HAN87_A1fi	6,202,392	
					HAN-87_A1se	3,133,702	Duplicate library
HAN-89	*Hanguana rubinea*	1.386°N	103.814°E		HAN-89_A1	3,336,487	
HAN-96	*Hanguana rubinea*	1.339°N	103.813°E	Genome size only			
HAN-85	*Hanguana* sp. 'Mandai'	1.407°N	103.785°E	Mixed population	HAN85_A1fi	6,086,960	**Cat***
					HAN85_A1se	6,907,432	Duplicate library **Cat***
HAN-25	*Hanguana* sp. 'MacRitchie'	1.352°N	103.822°E		HAN25_A1fi	5,334,721	**Cat***
					HAN25_A1se	3,815,381	Duplicate library **Cat***
HAN-34	*Hanguana* sp. 'MacRitchie'	1.350°N	103.813°E		HAN34_A1	4,779,680	**Cat***
HAN-03	*Hanguana triangulata*	1.350°N	103.778°E	Holotype locality	HAN03_A1	4,331,832	**Cat***
HAN-47	*Hanguana triangulata*	1.353°N	103.776°E		HAN47_F1	6,155,474	**Cat***
HAN-63	*Hanguana triangulata*	1.349°N	103.778°E		HAN63_A1	3,645,191	**Cat***
					HAN63_F3	4,079,497	
HAN-66	*Hanguana triangulata*	1.354°N	103.778°E	Mixed population	HAN66_A1fi	6,508,596	
					HAN66_A1se	3,829,805	Duplicate library
HAN-67	*Hanguana triangulata*	1.354°N	103.778°E		HAN67_A5fi	5,739,233	
					HAN67_A5Ase	5,427,631	Duplicate library
					HAN67_A5Bse	4,378,300	Duplicate library
HAN-69	*Hanguana triangulata*	1.356°N	103.777°E		HAN69_A2	5,958,615	
HAN-71	*Hanguana triangulata*	1.350°N	103.778°E		HAN71_A3	4,307,262	
HAN-72	*Hanguana triangulata*	1.348°N	103.778°E		HAN72_A1	6,271,274	
SING2020-584	*Cyrtostachys renda*	N/A	N/A	Cultivated, Singapore Botanic Gardens	SING2020_584	6,181,102	Outgroup
SING2020-586	*Pontederia cordata*	N/A	N/A	Cultivated, Singapore Botanic Gardens	SING2020_586	3,333,084	Outgroup
SING2020-588	*Tradescantia zebrina*	N/A	N/A	Cultivated, Singapore Botanic Gardens	SING2020_588	5,031,765	Outgroup
SNG291	*Zingiber singapurense*	N/A	N/A	Singapore, locality not released	SNG291	18,963,547	Outgroup

Supporting Information Table 2

Overview of holoploid (2C) genome sizes estimated in Singaporean *Hanguana* species.

Species	Mean 2C-DNA amount (pg)	Intraspecific variation (min/max *100%)	Ploidy	2C-DNA amount ± s.e. (pg)	Collection number
**Stoloniferous clade**					
Hanguana anthelminthica	2.29	0.7	4*x*	2.281 ± 0.3%<br>2.298 ± 0.3%	JLS-3033<br>JLS-3033
Hanguana nitens	1.276	4.3	2*x*	1.255 ± 0.6%<br>1.261 ± 0.6%<br>1.261 ± 0.9%<br>1.274 ± 0.9%<br>1.280 ± 0.7%<br>1.281 ± 0.5%<br>1.289 ± 0.5%<br>1.311 ± 1.1%	HAN-01<br>HAN-01<br>HAN-01<br>HAN-01<br>HAN-01<br>HAN-01<br>HAN-01<br>HAN-01
**Small forest species clade**					
Hanguana corneri	1.493	–	2*x*	1.493 ± 0.7%	HAN-04
Hanguana neglecta	3.561	3.6	5*x*	3.503 ± 0.2%<br>3.514 ± 0.4%<br>3.534 ± 0.5%<br>3.561 ± 0.6%<br>3.573 ± 0.5%<br>3.634 ± 0.3%	HAN-27<br>HAN-26<br>HAN-07<br>HAN-07<br>HAN-84<br>HAN-84
**Large forest species clade**					
Hanguana fraseriana	2.099	–	3*x*	2.083 ± 0.4%	FRI-89123
Hanguana podzolicola	2.05	1.3	3*x*	2.032 ± 0.0%<br>2.058 ± 0.2%<br>2.059 ± 1.0%	HAN-13<br>HAN-76<br>HAN-75
Hanguana rubinea	2.027	0.9	3*x*	1.990 ± 0.2%<br>2.009 ± 0.5%<br>2.012 ± 0.2%<br>2.012 ± 0.2%<br>2.019 ± 0.6%<br>2.027 ± 0.3%	HAN-73<br>HAN-10<br>HAN-70<br>HAN-96<br>HAN-02<br>HAN-96
Hanguana triangulata	2.001	–	3*x*	2.001 ± 0.3%	HAN-03
Hanguana sp. 'MacRitchie'	1.99	0.9	3*x*	1.981 ± 0.3%<br>1.999 ± 0.9%	HAN-34<br>HAN-25
Hanguana sp. 'Mandai'	1.995	2.4	3*x*	1.970 ± 0.1%<br>2.019 ± 0.6%	HAN-85<br>HAN-85

Supporting Information Figure 1

Effect of using multiple libraries made of different biological samples on relative contribution of a less frequent SNP to the total coverage in *Hanguana neglecta*. The position of the two observed peaks (20 % and 40 %, consistent with a pentaploid plant) was not affected by the se of multiple biological samples, but the signal improved with increased data. The result was expected, as the samples are considered clonal.

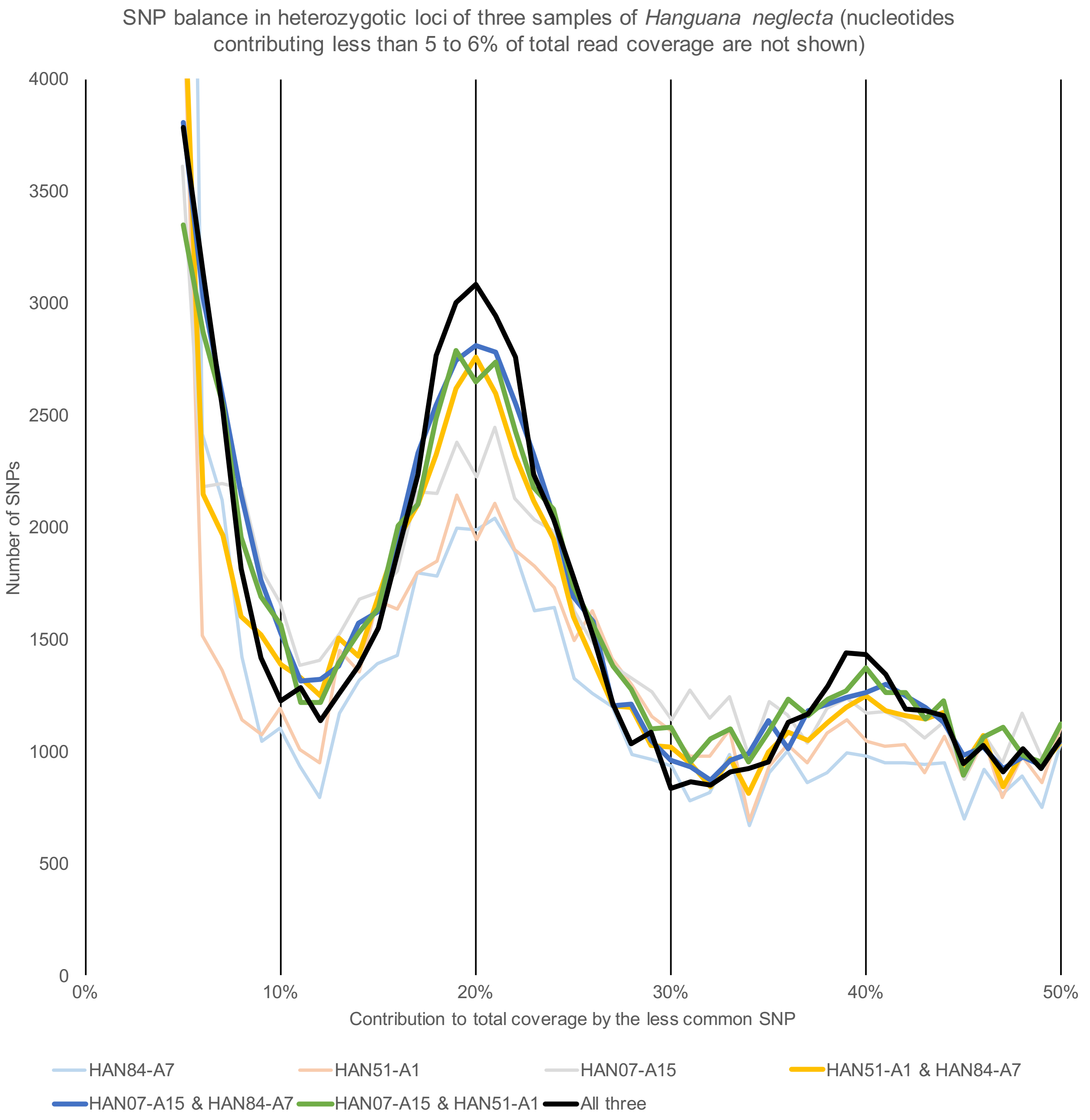

Supporting Information Figure 2

Pairwise overall SNP similarity of all samples sequenced in this study. This analysis was used to separate the samples to four sets with high similarity (>99.78 %) for population analyses. Separation of distantly related samples increased the available data for each group. *Hanguana nitens* and *H. anthelminthica* were both distinct from all other species and were analysed separately. 30 % missing data was allowed for each locus for a total of 23.511 SNPs.

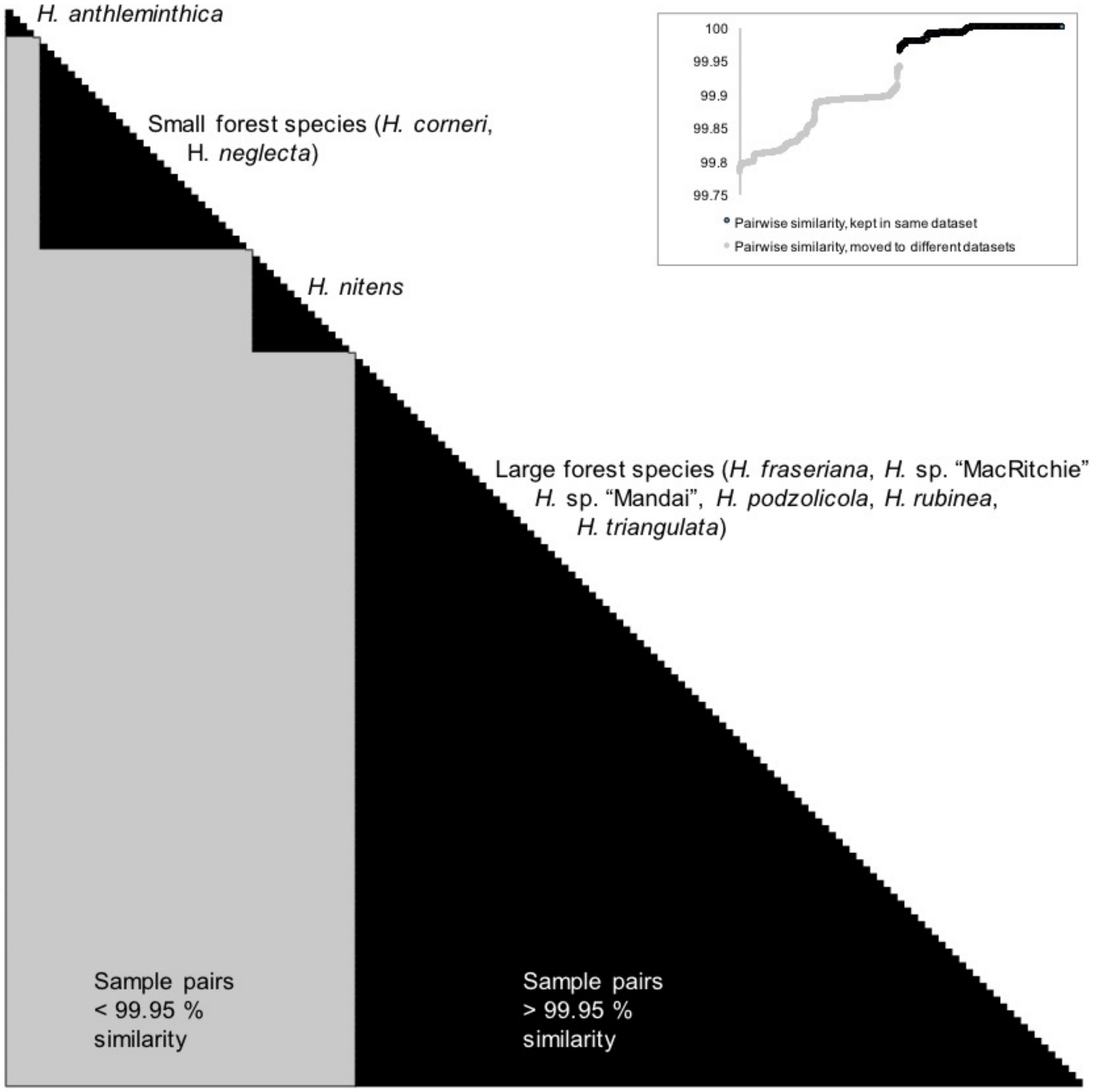

Supporting Information Figure 3

Identification of clonal pairs in all *Hanguana* datasets

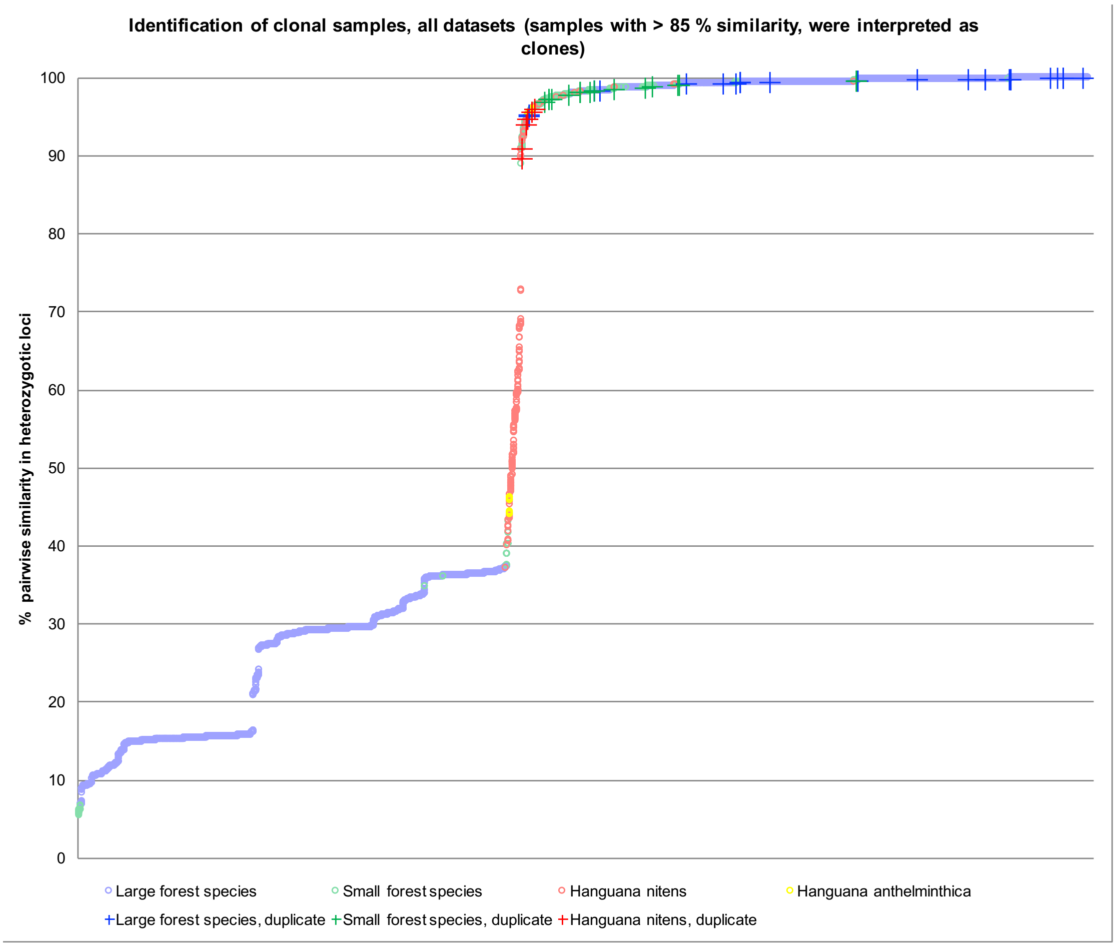

Supporting Information Figure 4

Rooted phylogenetic reconstruction, using closest outgroups (to species of Commelinaceae), other Commelinales (Pontederia) and further outgrops of commelinid monocots (Zingiberaceae and Arecaceae). Input data was ddRAD libraries that were mapped to consensus sequence of *Hanguana* samples, except for *Amischotolype hispi*da, for which we used whole genome sequencing data. (A) Topology with branch lengths transformed; (B) topology with original branch lengths; (C) topology with original branch lengths without outgroup.

A

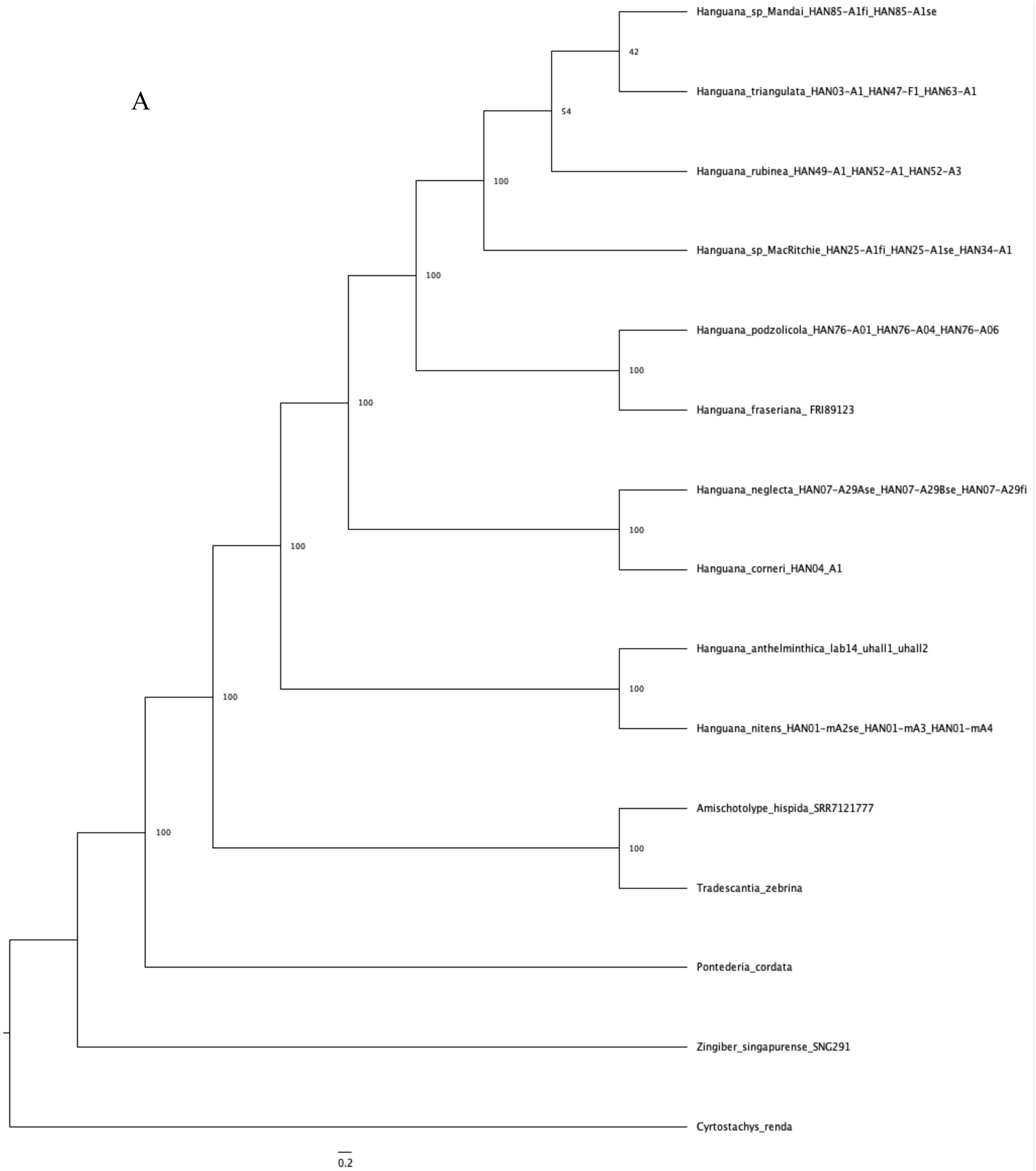

B

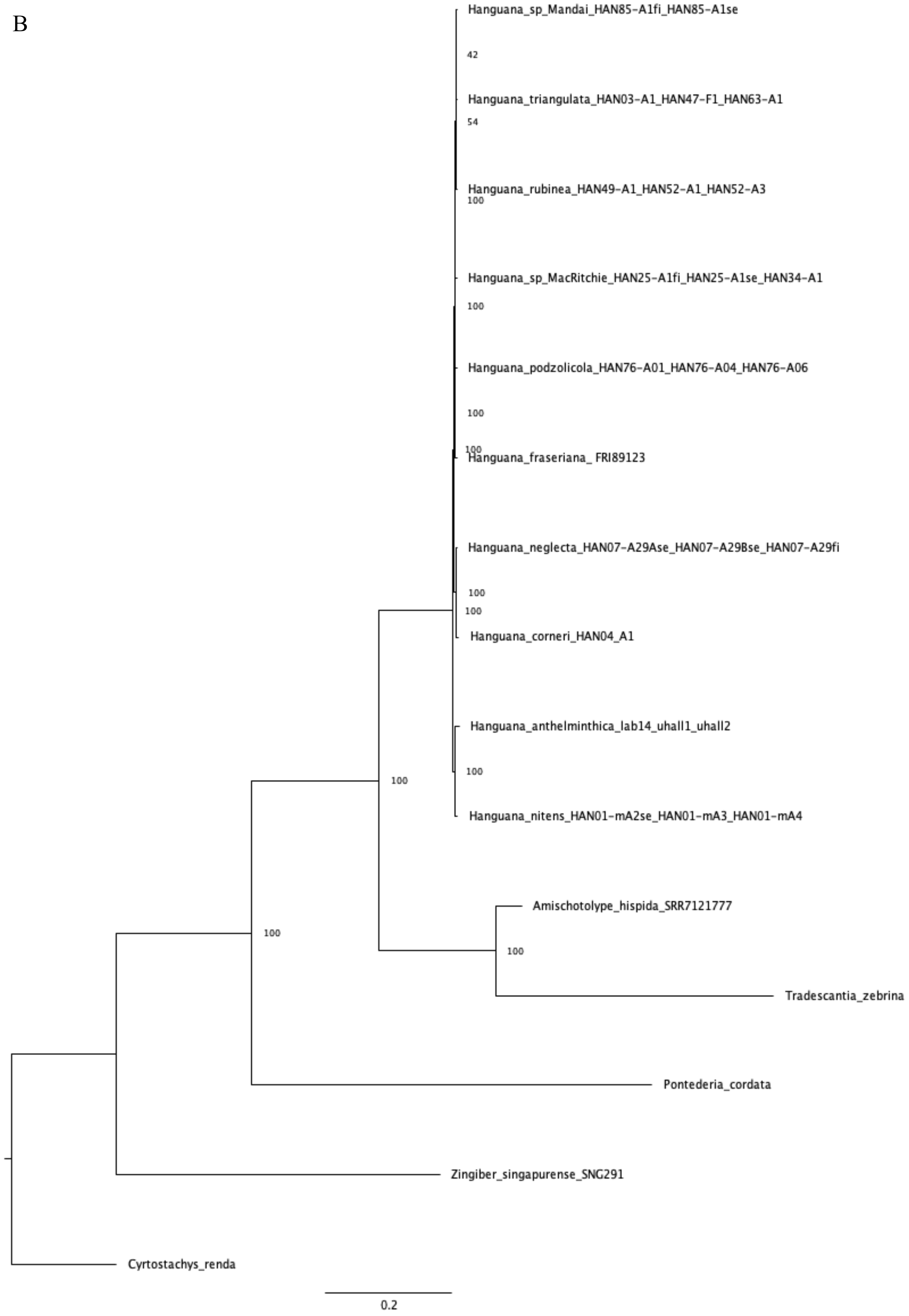

Hanguana_sp_Mandai_HAN85-A1fi_HAN85-A1se
42
Hanguana_triangulata_HAN03-A1_HAN47-F1_HAN63-A1
54
Hanguana_rubinea_HAN49-A1_HAN52-A1_HAN52-A3
100
Hanguana_sp_MacRitchie_HAN25-A1fi_HAN25-A1se_HAN34-A1
100
Hanguana_podzolicola_HAN76-A01_HAN76-A04_HAN76-A06
100
100
Hanguana_fraseriana_ FRI89123
Hanguana_neglecta_HAN07-A29Ase_HAN07-A29Bse_HAN07-A29fi
100
100
Hanguana_corneri_HAN04_A1
Hanguana_anthelminthica_lab14_uhall1_uhall2
100
100
Hanguana_nitens_HAN01-mA2se_HAN01-mA3_HAN01-mA4
Amischotolype_hispida_SRR7121777
100
100
Tradescantia_zebrina
Pontederia_cordata
Zingiber_singapurense_SNG291
Cyrtostachys_renda
0.2

C

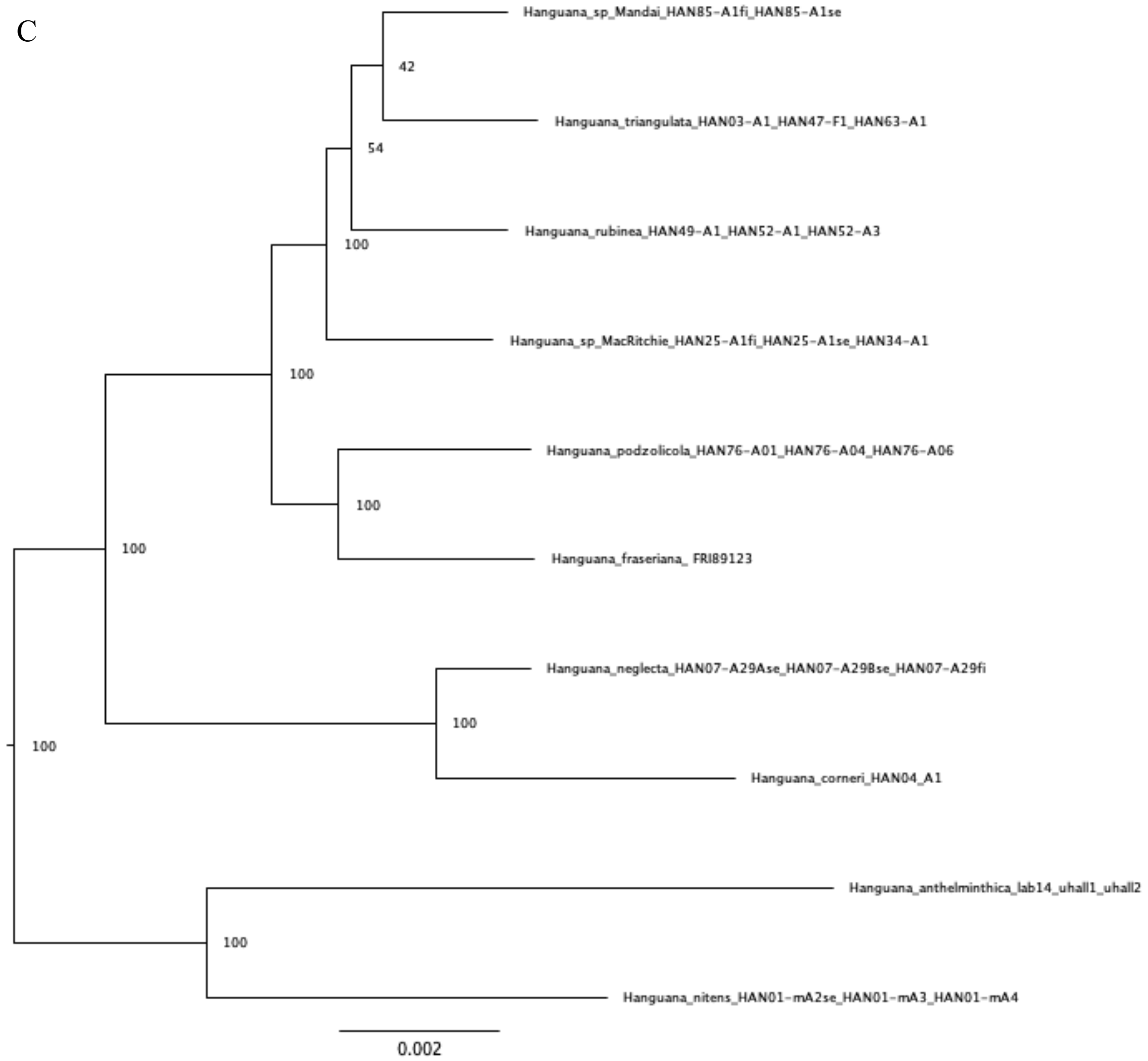
Hanguana_sp_Mandai_HAN85-A1fi_HAN85-A1se
42
Hanguana_triangulata_HAN03-A1_HAN47-F1_HAN63-A1
54
Hanguana_rubinea_HAN49-A1_HAN52-A1_HAN52-A3
100
Hanguana_sp_MacRitchie_HAN25-A1fi_HAN25-A1se_HAN34-A1
100
Hanguana_podzolicola_HAN76-A01_HAN76-A04_HAN76-A06
100
100
Hanguana_fraseriana_ FRI89123
Hanguana_neglecta_HAN07-A29Ase_HAN07-A29Bse_HAN07-A29fi
100
100
Hanguana_corneri_HAN04_A1
Hanguana_anthelminthica_lab14_uhall1_uhall2
100
Hanguana_nitens_HAN01-mA2se_HAN01-mA3_HAN01-mA4
0.002

Supporting Information Figure 5

Chromosome number variation in *Hanguana*; A) *Hanguana corneri*, 2*n* = ~ 40–48; B) *Hanguana nitens*, 2*n* = ~ 40–48; C) *Hanguana rubinea*, 2*n* = ~ 60–72; D) *Hanguana podzolicola*, 2*n* = ~ 60–72; E) *Hanguana anthelminthica*, 2*n* = ~ 80–96; F) *Hanguana neglecta*, 2*n* = ~ 120–144.

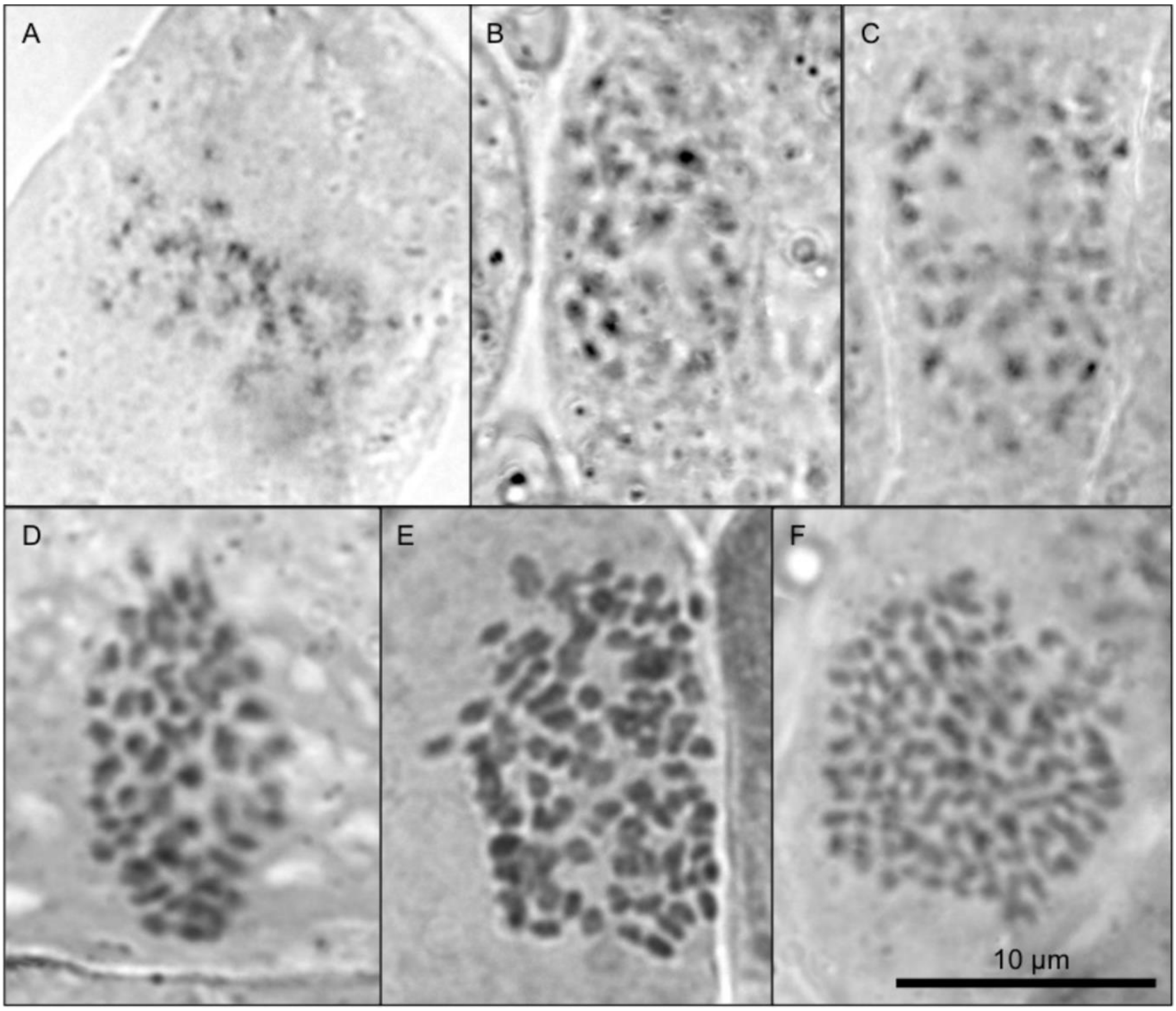

Supporting Information Figure 6

Representative flow cytometric histogram documenting genome size determination in *Hanguana rubinea* (accession number HAN-73) using *Solanum pseudocapsicum* as internal reference standard.

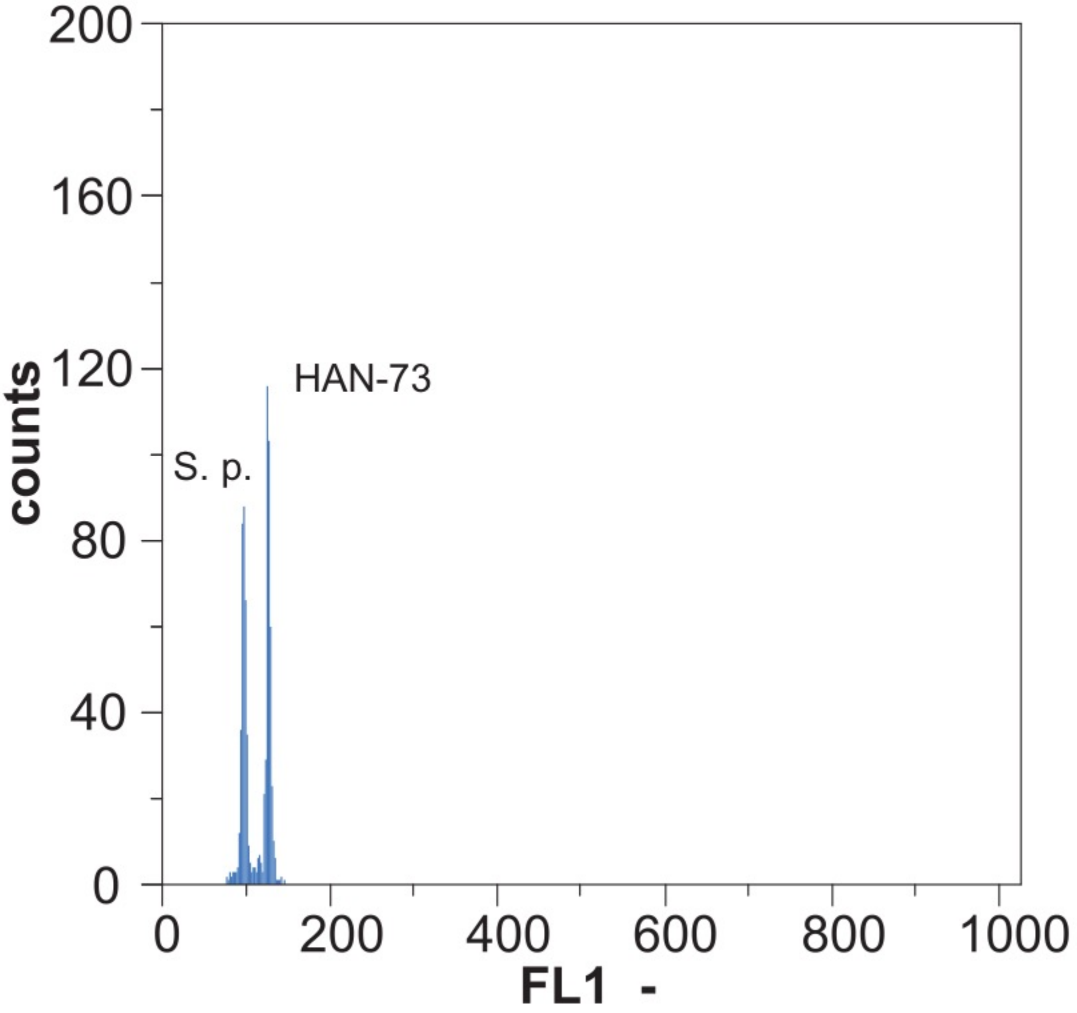

Supplement: plaa053_suppl_Supplementary_Materials [file plaa053_suppl_supplementary_materials.pdf]
